# Correctness is its own reward: bootstrapping error signals in self-guided reinforcement learning

**DOI:** 10.1101/2025.07.18.665446

**Published:** 2025-08-19

**Authors:** Ziyi Gong, Fabiola Duarte, Richard Mooney, John Pearson

**Affiliations:** 1 Department of Neurobiology, Duke University, Durham, NC, USA; 2 Department of Cell Biology, Duke University, Durham, NC, USA; 3 Department of Electrical and Computer Engineering, Duke University, Durham, NC, USA

## Abstract

Reinforcement learning (RL) offers a compelling account of how agents learn complex behaviors by trial and error, yet RL is predicated on the existence of a reward function provided by the agent’s environment. By contrast, many skills are learned without external guidance, posing a challenge to RL’s ability to account for self-directed learning. For instance, juvenile male zebra finches first memorize and then train themselves to reproduce the song of an adult male tutor through extensive practice. This process is believed to be guided by an internally computed assessment of performance quality, though the mechanism and development of this signal remain unknown. Here, we propose that, contrary to prevailing assumptions, tutor song memorization and performance assessment are subserved by the same neural circuit, one trained to predictively cancel tutor song. To test this hypothesis, we built models of a local forebrain circuit that learns to use contextual input from premotor regions to cancel tutor song auditory input via plasticity at different synaptic loci. We found that, after learning, excitatory projection neurons in these circuits exhibited population error codes signaling mismatches between the tutor song memory and birds’ own performance, and these signals best matched experimental data when networks were trained with anti-Hebbian plasticity in the recurrent pathway through inhibitory interneurons. We also found that model learning proceeds in two stages, with an initial phase of sharpening error sensitivity followed by a fine-tuning period in which error responses to the tutor song are minimized. Finally, we showed that the error signal produced by this model can train a simple RL agent to replicate the spectrograms of adult bird songs. Together, our results suggest that purely local learning via predictive cancellation suffices for bootstrapping error signals capable of guiding self-directed learning of natural behaviors.

## 1 Introduction

The ability to acquire and maintain skilled motor behaviors is among the most impressive capabilities humans possess, from hitting a major league baseball to performing a Bach concerto. Yet learning these highly refined skills requires extensive practice, typically without the aid of external rewards or punishments. As part of this process, learners must therefore assess their own progress, presumably through a comparison of sensory feedback with some desired state. That is, for self-guided reinforcement learning to be possible, agents must somehow construct an internal reward function, a representation of high-quality performance capable of guiding subsequent behavioral adjustments.

In nature, the song copying behavior of male zebra finches provides a sophisticated example of precisely this type of learning. Early in development, juveniles memorize the courtship song of an adult male tutor during an initial “sensory” phase of learning, using it to guide independent practice in the subsequent “sensorimotor” phase, with the goal of replicating the tutor song (Roberts & Mooney, 2013). This copying process has typically been understood through the framework of reinforcement learning (RL) (R. Chen & Goldberg, 2020; Doya & Sejnowski, 1998; Fee & Goldberg, 2011; Sutton & Barto, 2018). In RL, animals alter their behaviors over time to gain rewards or avoid punishments, operationalized as maximizing the total reward delivered by the environment. In multiple species, such learning is known to be subserved by dopaminergic neurons in the ventral tegmental area (VTA), whose responses signal the mismatch between actual and expected reward (Gershman & Uchida, 2019; Gershman et al., 2024; Markowitz et al., 2018, 2023; Reynolds & Wickens, 2002; Schultz, 2007; Tsai et al., 2009; Yagishita et al., 2014). Likewise, dopamine has also been shown to be a critical driver for song learning in zebra finches (Gadagkar et al., 2016; Hisey et al., 2018; Hoffmann et al., 2016; Kasdin et al., 2025; Qi et al., 2025; Xiao et al., 2018), though in this case, it is an *internally-generated* reinforcement signal tracking performance quality relative to some standard (R. Chen & Goldberg, 2020; Doya & Sejnowski, 1998; Gadagkar et al., 2016; Kasdin et al., 2025; Qi et al., 2025). The fact that this standard — a particular tutor song — is not innate but learned thus raises the question of how juveniles bootstrap these performance errors.

Previous studies have implicated multiple interconnected auditory nuclei in signaling errors in birds’ own songs (Bolhuis & Moorman, 2015; Keller & Hahnloser, 2009; Mandelblat-Cerf et al., 2014), including regions projecting to VTA (R. Chen et al., 2019; Kearney et al., 2019; Keller & Hahnloser, 2009; Mandelblat-Cerf et al., 2014). Hypothetically, a neuron representing evaluative information should deviate from its baseline activity when sensory feedback during singing differs from desired behavior, while its activity should remain the same when performance meets expectations or the bird is silent. Indeed, certain neurons from Field L, caudolateral mesopallium (CLM) and ventral intermediate acropallium (Aiv) in adult birds respond to disruptive auditory feedback presented during singing but not to uninterrupted singing nor playback of normal or distorted song during non-singing periods (Keller & Hahnloser, 2009; Mandelblat-Cerf et al., 2014). Moreover, this error information is likely to converge in the intermediate arcopallium (Aiv) (Mandelblat-Cerf et al., 2014), since Aiv projects directly to VTA and pitch-dependent optogenetic activation of Aiv → VTA terminals results in negative reinforcement of syllable pitch in adult birds (Kearney et al., 2019).

Another important finding is that several of these auditory regions directly or indirectly receive premotor inputs from area HVC (proper name) (Akutagawa & Konishi, 2010; Mandelblat-Cerf et al., 2014; Nottebohm et al., 1982; Roberts et al., 2017). The presence of sensorimotor inputs to sensory regions suggests the possibility of corollary discharge mechanisms for the cancellation of self-generated sensory input, similar to those observed in mice (Harmon et al., 2024; Schneider et al., 2014, 2018), primates (Crapse & Sommer, 2008), and weakly electric fish (Dempsey et al., 2019; Kennedy et al., 2014; Wallach & Sawtell, 2023). Likewise, there is evidence in adult birds that premotor input from HVC to auditory regions may govern the selective engagement of error-responding neurons during singing (R. Chen & Goldberg, 2020; Kearney et al., 2019; Mandelblat-Cerf et al., 2014). Interestingly, HVC neurons have been found to exhibit tutor-song responsiveness in awake birds at early ages (Adret et al., 2012; Mackevicius et al., 2020; Moseley et al., 2017; Nick & Konishi, 2005; Schroeder & Remage-Healey, 2024; Vallentin et al., 2016), a feature which declines with age (Nick & Konishi, 2005) and disappears in adults (Nick & Konishi, 2001; Schmidt & Konishi, 1998). Thus, both the sensory and premotor information necessary for computing performance errors are present directly upstream of VTA, though the mechanism by which vocal performance error is computed remains unknown.

**Figure 1:**
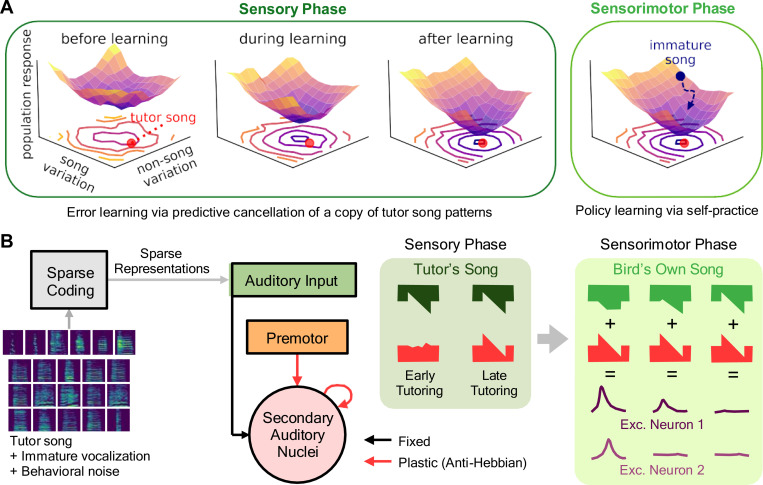
Learning to cancel tutor song results in an error landscape sufficient for subsequent motor learning. **(A)** Schematic of the general hypothesis. Neuronal responses to sensory input define a multi-dimensional, time-dependent “error landscape.” During the sensory learning phase, local learning rules increase the landscape’s curvature (sensitivity to error) and move its minimum toward the target tutor song (red dot). In the subsequent sensorimotor learning phase, this error serves as a negative reward, and reinforcement learning algorithms refine the bird’s own song by performing error minimization on this landscape. **(B)** General model structure. A sparse coding model maps tutor song to auditory input, which is sent on to meet song-locked premotor timing input in secondary auditory nuclei. Local plasticity rules in these regions gradually learn to perform predictive cancellation of this pattern, giving rise to sparse error codes.

Here, inspired by these corollary discharge ideas, we hypothesize that during the sensory phase of learning, local learning rules in auditory areas of juvenile finches facilitate predictive cancellation of a copy of tutor song using cues provided by premotor inputs. That is, we propose that tutor song memorization and performance error computation are not separate processes but subserved by a single circuit mechanism. As a result, projection neurons in these areas gradually form a sparse population error code sufficient for guiding reinforcement learning in the sensorimotor phase (Fig. 1A). To test this possibility, we implemented a set of models varying in circuit structure, locus of plasticity, and type of learning rule, comparing against existing data recorded in auditory nuclei. We found that a balanced excitatory-inhibitory network with anti-Hebbian plasticity in the recurrent connections involving interneurons was most consistent with experimental data. Moreover, in analyzing the learning dynamics of this network, we found that the “error landscape” — the geometry of population responses — exhibits two phases during the sensory learning period: During the first phase, the gain of error responses increases, sharpening the error landscape. In the second phase, the minimum of this landscape moves to align with the tutor song. Lastly, we found that, using the error codes acquired by fitting to real zebra finch song data, we could train a simple agent to accurately copy a tutor song spectrogram via reinforcement learning. Together, these findings show that purely local learning mechanisms for predictive cancellation are sufficient for estimating multidimensional performance errors, signals sufficient to enable self-guided learning of natural behaviors.

## 2 Results

To test for the emergence of error codes via local learning, we implemented a simplified model of the interconnected auditory areas of the forebrain as a single network receiving two inputs: upstream auditory input and premotor input (Fig. 1B). For the upstream auditory input, we assumed a sparse encoding of the song spectrogram (Schneider & Woolley, 2013; Smith & Lewicki, 2006) and trained a classic sparse coding model (Olshausen & Field, 1996) using a combination of adult male zebra finch songs (Koch, 2024), immature vocalizations (Brudner et al., 2023), and noise caused by behaviors such as flapping. The outputs of this model were then used as the firing rates of the auditory input population (Fig. S1; see Methods).

For premotor input, we assumed a sparse, sequential activity pattern in which individual units burst at well-defined moments during the song, similar to observed activity in HVC (Hahnloser et al., 2002; Long et al., 2010). While this pattern is well-documented in adult finches, it has also been observed that HVC contains tutor-song-selective neurons at early ages (Adret et al., 2012; Moseley et al., 2017; Nick & Konishi, 2005; Schroeder & Remage-Healey, 2024; Vallentin et al., 2016). However, to test the sensitivity of our results to the precision of this premotor sequence, we also replicated our experiments using a “developing” premotor input in which the peak time and peak width of premotor input are initially strongly irregular and only gradually become regular over learning. We found that using such premotor input for training does not affect the ability of the network to learn error codes that match experiments (Fig. S2; see below).

**Figure 2:**
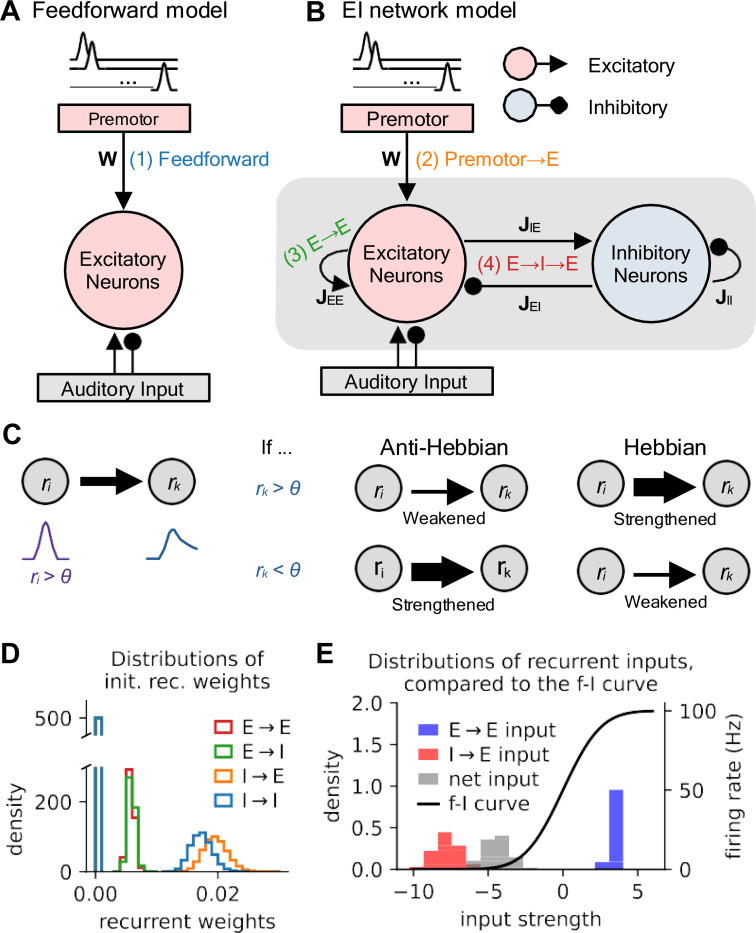
Four classes of models for local learning. **(A)** Feedforward model. Song-related premotor input directly drives excitatory neurons in secondary auditory areas, which receive both excitatory and inhibitory input from primary auditory areas. Plasticity takes place at the premotor→secondary auditory synapse (1). **(B)** Balanced excitation-inhibition (EI) networks with three potential sites of plasticity (2–4). As in **(A)**, excitatory neurons in secondary auditory areas receive input from both primary auditory areas and premotor areas, as well as a local population of inhibitory interneurons. Numbers indicate the site(s) of plasticity in each of the three models. **(C)** Schematic of rate-based Hebbian and anti-Hebbian plasticity rules. Hebbian rules strengthen synaptic connections when postsynaptic excitation exceeds a threshold θ. Anti-Hebbian rules do the reverse. **(D)** Distributions of initial recurrent weights for the four types of connections. The bins at zero represent the 50% of connections in each model type that are set to 0 for sparsity. In general, inhibitory weights need to be stronger than excitatory weights to achieve EI balance. A detailed description of the parameters and construction of weights is in Methods and Table 1. **(E)** Illustration of EI balance. Left vertical axis: density of the distributions of E→E (blue), I→E (red), and their net inputs (gray); right vertical axis: output firing rate of the f-I curve (black). EI balance is achieved when the distribution of net inputs lies near the activation threshold of the f-I curve.

We modeled the local auditory circuit as consisting of a single population of excitatory projection neurons receiving excitatory premotor input and both excitatory and inhibitory sensory input from the sparse coding network. In other models, we added to this excitatory population a reciprocally connected pool of local inhibitory interneurons. To test the effect of different types of local learning rules and synaptic loci, we considered four models: (1) A feedforward network of excitatory neurons, with anti-Hebbian plasticity in premotor → E connections (Fig. 2A) and three balanced excitation-inhibition (EI) networks of interconnected excitatory (E) and inhibitory (I) neurons (Fig. 2B) with differing loci of learning: (2) anti-Hebbian premotor → E connections; (3) anti-Hebbian E → E connections; or (4) *Hebbian* E → I and I → E connections^†^. That is, in Hebbian plasticity models, a synapse was strengthened (or weakened) if the pre- and postsynaptic neurons were co-active (or not co-active) (Fig. 2C), while for anti-Hebbian models, the contingency was reversed. For simplicity, we implemented this learning using bilinear plasticity rules (Brown et al., 1990).

To achieve excitatory-inhibitory balance, we initialized the recurrent connection weights of the EI networks to be strong and random, with means obeying the balanced condition (van Vreeswijk & Sompolinsky, 1996) (Fig. 2D), such that the net recurrent input to a neuron lay within the linear regime of its activation function, even if recurrent excitation or inhibition would separately have produced runaway or silent activity, respectively (Fig. 2E). Together, these models produced sparse activity at biologically plausible firing rates while allowing us to consider multiple possibilities for both circuit connectivity and the locus of synaptic plasticity during learning.

### 2.1 Learning predictive cancellation enables neurons to signal error

**Figure 3:**
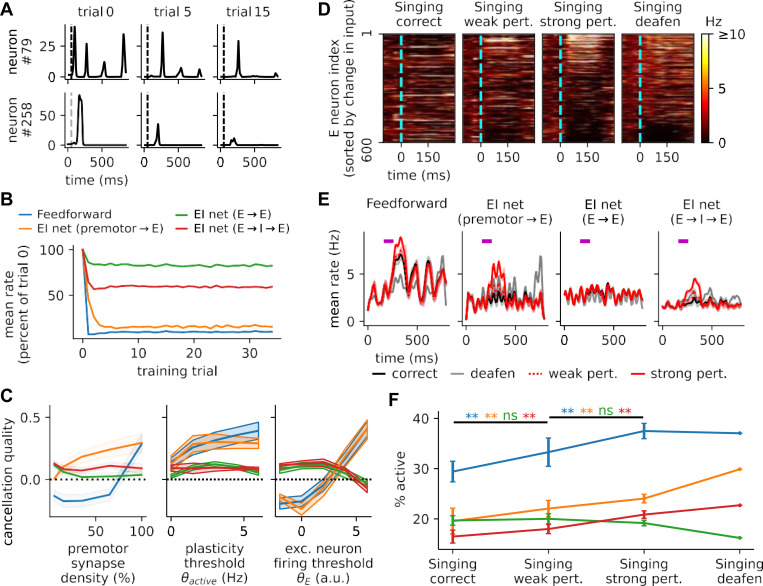
Neurons produce sparse error codes after learning to cancel tutor song auditory patterns. **(A)** Two example neurons in the E→I→E model decrease firing over learning. Gray dashed vertical lines mark song onset. Other models are qualitatively similar. **(B)** Population mean firing rates decrease over training, relative to the initial population means. Feedforward and non-recurrent models exhibit the largest percent decreases. **(C)** Quality of cancellation (see Methods) in the four models as functions of premotor projection density, plasticity threshold $\theta_{\text{active}}$, and excitatory neuron activation threshold $\theta_{E}$. Positive (or negative) quality of cancellation indicates lower (or higher) similarity of population activity with tutor song patterns after training. Widths of the shaded areas denote 1 s.d. across 20 initializations of each model. Models with recurrent plasticity perform best in the physiological regime of sparse inputs and low firing thresholds. **(D)** Learned singing responses under practice and perturbation. Raster plots show the responses of all excitatory neurons during singing under conditions of correct song (matching the tutor song), weak perturbation (small added noise), strong perturbation (strong added noise), and deafening (no auditory input). Neurons are sorted by the difference between auditory input (bird’s own song) patterns and the sample-averaged tutor song pattern. **(E)** Population mean rates in the correct (black), deafened (grey), weak perturbation (dotted red), and strong perturbation (solid red) cases during singing. The purple bar in each subplot indicates the 100-ms white noise perturbation to the auditory feedback of the bird’s own song. Only the E→ E model fails to exhibit a population response to error. **(F)** Percentages of active excitatory neurons ($\bar{r}\geq 3\;\text{Hz}$, see Methods) in different models, averaged across all samples, from the earliest perturbation onset to 100 ms after the latest perturbation offset. Except for the E→ E model, a denser set of neurons are active during perturbed singing than during correct singing. Note that the learning rate is set to zero in **(D-F)**.

We simulated the sensory phase of learning by training each of our models to cancel sparse auditory embeddings of the spectrograms of real adult male zebra finch songs (Fig. 1B). As expected, anti-Hebbian learning reduced the activity at both the population and individual neuron levels (Fig. 3A-B), and plastic weights projecting to the excitatory neurons of auditory regions were more negatively correlated with tutor song input after learning (Fig. S3), allowing local input to cancel the expected auditory pattern of the tutor songs.

Comparing across models, we found that while firing rate reductions were more pronounced in the feedforward and premotor→E models (Fig. 3B), the quality of tutor song cancellation depended on multiple parameters. In particular, we probed how the cancellation depended on three important and experimentally measurable quantities: the density of premotor synapses, the equilibrium threshold of plasticity, and the excitatory neuron firing threshold (Fig. 3C). When premotor projection density is sparse, as observed in experiments (Mandelblat-Cerf et al., 2014; Roberts et al., 2017), cancellation is more effective in the E → E and E → I→ E models than the models with premotor→ E plasticity, because the latter models must directly store negative images of tutor song patterns in the limited premotor→ E weights. In this sparse premotor projection scenario, the error codes in the feedforward and premotor→ E models are degraded (Fig. S4). Furthermore, the quality of cancellation remains relatively stable over different plasticity thresholds across all models, while for the excitatory neuron firing threshold, the feedforward and premotor→ E models fail to cancel tutor song patterns unless the excitatory neurons are hard to activate, while the E → E and E → I→ E models favor intermediate to low thresholds (Fig. 3C). Thus, models with recurrent plasticity are more robust to different parameter values in our models.

Having verified that models could reliably cancel tutor song, we then proceeded to assess their error-coding capacity by testing their responses to unanticipated auditory inputs. Following experiments in which the responses of auditory neurons were probed by playing either undistorted or noise-corrupted recordings of the bird’s own song (Keller & Hahnloser, 2009; Mandelblat-Cerf et al., 2014), we set the learning rates of our fully-trained models to zero and observed their responses under these conditions. To simulate the correct singing case, in which the bird’s own song matches the tutor song, we randomly selected samples from tutor song data as the auditory inputs. In response to these stimuli, most excitatory neurons in a local forebrain circuit exhibited low transient activity (Fig. 3D). In contrast, when the auditory feedback of the bird’s own song was perturbed by directly adding 100ms of white noise to the stimulus, these neurons displayed heterogeneous error responses, with larger firing rates on trials with larger mismatch between auditory feedback and the sample-averaged tutor song. This finding was also replicated with stronger white noise perturbation. In addition, when the auditory feedback was removed, simulating the case of singing in deafened birds, neurons weakly signaled the *inverse* pattern of tutor song—indicative of predictive cancellation. At the population level, the mean rates (Fig. 3E) and percentages of active neurons within the perturbation time window (Fig. 3F) of the excitatory population were increased by perturbation and deafening in all but the E → E model. Though the E → E model did not exhibit a mean population error response, it nonetheless showed heterogeneous error responses like the others (Fig. S5A-D).

We also observed that increases in population mean firing rates and percentages of active neurons did not persist to the end of the song (Fig. 3E and Fig. S5E), agreeing with experimental findings (Mandelblat-Cerf et al., 2014). Using perturbations much shorter than the duration of a syllable, we further verified that the circuits can signal error on a sub-syllabic basis (Fig. S6A-C). Together, these results suggest that, simply by using local learning to cancel tutor song, our models simultaneously developed sparse population error codes with high temporal precision.

### 2.2 Experimental data favor error codes learned via inhibitory plasticity

**Figure 4:**
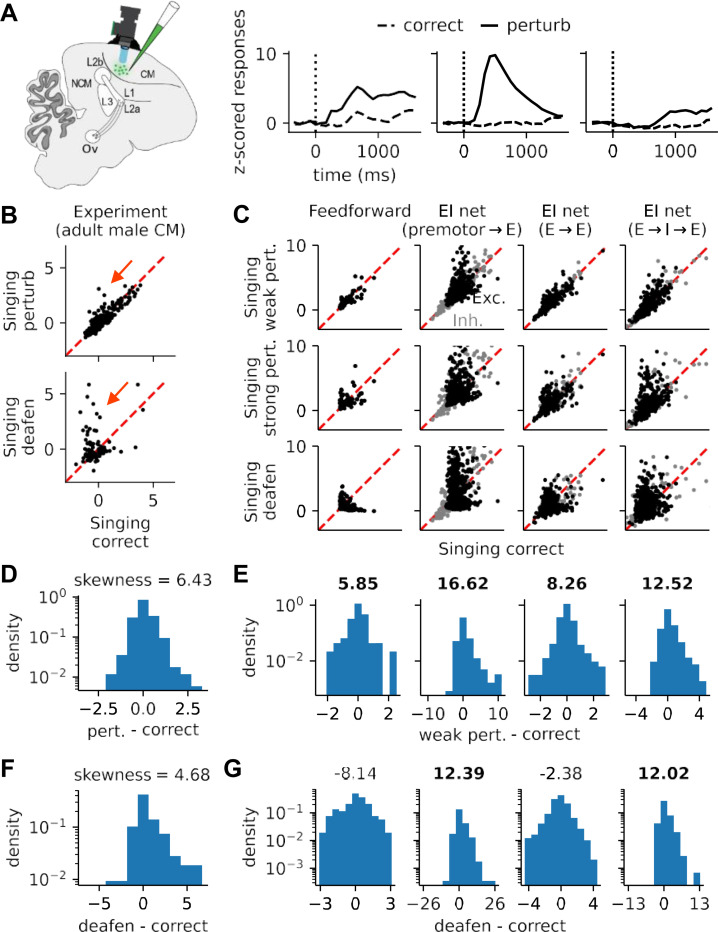
Comparison of experimental data to models with different sites of local plasticity. **(A)** Left: schematic of the pan-neuronal calcium imaging experiments. Right: z-scored responses of three example units in the case of correct singing (dashed lines) and white noise perturbation (solid lines). **(B)** Distributions of trial-averaged z-scored calcium fluorescence in correct versus perturbed singing in area CM of adult male zebra finches. Top row: scatterplot of white noise perturbation responses versus responses on correct singing trials. A sparse set of neurons are more activated by white noise perturbation (red arrow). Each dot represents the trial average of the normalized activity of one neuron over the song period (see Methods). Bottom row: same as top row, but comparing responses post-deafening with correct singing responses pre-deafening. **(C)** Same as **(B)** for the four classes of plasticity models (with model firing rates instead of fluorescence). Black dots are excitatory neurons and grey dots inhibitory neurons. In the models, only excitatory neurons exhibit the sparse error responses seen the experimental data, with some qualitative differences across models. **(D-G)** Distributions of the differential neuronal responses between correct singing and either perturbation **(D,E)** or deafening **(F,G)**. **(D,F)** show these values for data, and **(E,G)** for models. For the models, both excitatory and inhibitory neurons are included. Numbers above each histogram indicate the skewness of the distribution. All distributions are significantly skewed (*p* < 0.01, two-sided Wilcoxon signed-rank test). For **(E)**, data from simulations of weak noise perturbation in the models were used for the comparison. Strong perturbations produce even more skewed distributions, resulting in identical conclusions.

As stated above, our models considered four potential kinds of local plasticity in auditory circuits. To determine which of these candidate model is most likely, we compared the error codes from our models with neural activity obtained from one-photon calcium imaging performed in the caudal mesopallium (CM) of adult male zebra finches (Fig. 4A) before and after deafening. CM is a primary auditory region and also receives input directly from the premotor area HVC. During recording, we used a closed-loop system (Tumer & Brainard, 2007) to randomly target 50% of syllable renditions with white noise to induce an artificial vocal error. We found that CM neurons displayed sequential activation or suppression during singing, with this activity disrupted by white noise perturbation (Fig. S7A). In a sparse set of neurons, mean responses to these perturbations increased relative to correct singing, while a larger subset of neurons increased their responses during song post-deafening (Duarte Ortiz, 2024) (Fig. 4B).

Our models replicate these effects. We found both intact and disrupted sequential activation during normal and perturbed singing, respectively, though only the E → E and E → I→ E models could additionally produce the observed sequential suppression (Fig. S7B). Moreover, in comparing perturbed to unperturbed responses, we found a sparse set of neurons more activated by white noise perturbation and deafening than during normal singing, and such activation is stronger and denser in the deafening case than white noise perturbation (Fig. 4C). In our models, this effect was driven by excitatory but not inhibitory neurons.

Yet, while all models contained such a sparse population of perturbation-responsive neurons, not all models provided an equally good quantitative match to data. To quantify the prevalence and magnitude of this perturbation response across models, we computed the distribution of differential neuronal responses across both the white noise (perturbed minus correct; Fig. 4D-E) and deafened (deafened minus correct; Fig. 4F-G) conditions. We found that the experimental distributions of these effects were strongly right-skewed (Fig. 4D and F), an observation reproduced by all models in the perturbation condition and only the premotor→E and E→I→E models in the deafened condition. Furthermore, the premotor→ E model was unable to reproduce this effect when premotor input was sparse (Fig. S4) or highly irregular during training (Fig. S2D-E), while the E → I → E model reliably reproduced the experimental observations in those more challenging and realistic scenarios. A brief summary of the results of these simulations is provided in Table 1, which suggests that the E → I → E model with local inhibitory plasticity best matches experimental data.

### 2.3 Population error codes exhibit two-stage learning

**Figure 5:**
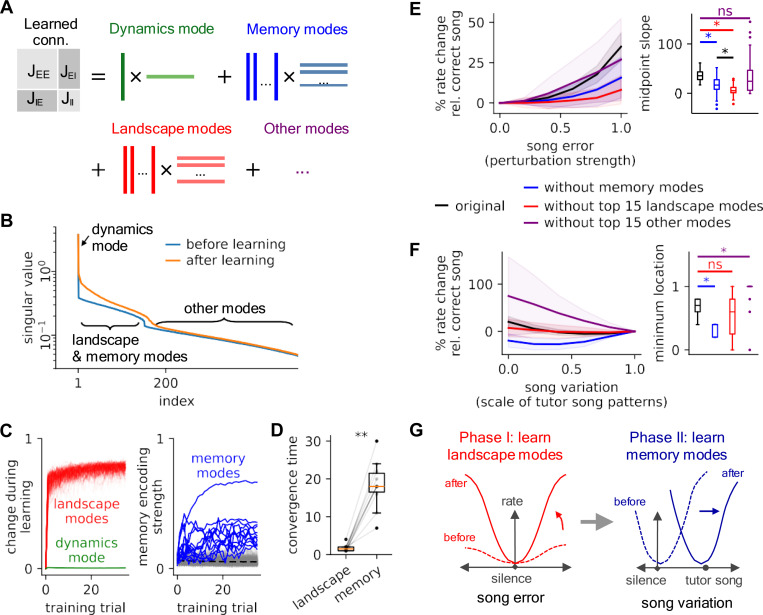
The minimum and shape of the error landscape are encoded by different connectivity modes in the E→ I →E model. **(A)** Schematic of the singular value decomposition of the concatenated connectivity matrix. **(B)** The sorted singular value spectra before (blue) and after (orange) learning. The spectrum exhibits a clear kink that we use to differentiate between dynamics, landscape, and memory modes and “other modes.” **(C)** Left: Dissimilarity between the non-memory modes and their initial values over training time. The green curve is the dynamic mode, which barely changes, and the red curves are landscape modes, which change rapidly and quickly stabilize. Right: Strength of memory encoding for all modes during training. The blue curves are those with final memory encoding strengths larger than the maximum pre-learning memory encoding strength (i.e., noise correlation). **(D)** Convergence time for landscape modes and memory modes (asterisk indicates *p* < 0.01, one-sided Wilcoxon signed-rank test), quantifying the time trends shown in **(C)**. **(E, F)** Altering landscape and memory modes alters population error responses. Relative responses are defined as the percentages of population rate change compared to the correct singing responses. Comparisons among the original trained models (black); models with the top 15 landscape modes removed (red), with memory modes removed (blue); and with the top 15 non-memory, non-landscape modes removed (purple). Asterisks in the the right panels indicate significant differences between distributions (*p* < 0.01; two-sided Wilcoxon signed-rank test; colored asterisks: altered model versus original model; black asterisks: models with perturbed memory versus landscape modes). **(E)** Left: model response as a function of perturbation strength (0: correct song; 1: random embedding). Altered landscape modes show lower response gains. Right: the midpoint slope is the slope of the line tangent to the curves at 0.5 on the left plot. **(F)** Left: rate changes as a function of song scaling (0: deafening; 1: correct song). Minimum network responses shift when the memory modes are altered. Right: the locations of the minima of the curves. Perturbations of the landscape modes primarily affect the error landscape slope, while perturbations of the memory modes alter its minimum. **(G)** Schematic of changes to the error landscape as a result of learning.

In our models, local learning rules give rise to predictive cancellation of tutor song inputs, producing as a byproduct sparse population error codes. Viewed as a function of auditory input, these codes describe an “error landscape” whose geometry might guide subsequent reinforcement learning. To understand how these codes develop at the population level, we analyzed the spectrum of the recurrent weight matrix J of our most plausible candidate, the E → I → E model, over the course of training. Since this matrix governs recurrent population dynamics in response to auditory input, understanding its changes during learning sheds light on the process by which the error landscape takes shape. Specifically, we analyzed J using its singular value decomposition (SVD), decomposing it into a sum of rank-1 input-output pattern pairs (Fig. 5A-C). Each of these pairs describes a “singular mode,” an independent dynamical mode in population responses.

We found that, while J remains high-rank throughout learning (Fig. 5B), its singular modes are readily separable into groups based on their learning time courses and effects on the error landscape. In all cases, the top singular mode of J was unchanged by learning (Fig. 5B-C) and encoded the network’s basic connectivity structures and responses to transients; perturbing it resulted in runaway dynamics (Fig. S8). Since this mode ensures the dynamic balance of neural activity, we refer to it as the “dynamic mode.” After the dynamic mode, we found range of singular modes that changed significantly over the course of learning (Fig. 5B-C). Among these, a small number (N≤15) eventually developed correlations with tutor song patterns, while a second group that did not exhibit this pattern nonetheless changed rapidly and quickly stabilized after learning began. For reasons that will become clear below, we will refer to these as groups as “memory modes” and “landscape modes,” respectively. Finally, beyond these groups remained a long tail of modes (“other modes”) with much smaller singular values that we did not analyze. In terms of time course, we found that the landscape modes exhibited fastest learning, as measured by the divergence between their start and end patterns, followed by the memory modes, whose magnitudes of correlation with tutor song patterns all grew over the course of training, albeit more slowly (Fig. 5D).

How do these distinct classes of modes affect the learned error landscape? To understand this phenomenon, we performed perturbation experiments in which we altered one or both of sets of modes in the network weights post-learning. Specifically, we either (1) “de-memorized” the models by removing the correlation between memory modes and tutor song patterns*, or (2) shuffled the entries of a similar number of landscape modes or (3) shuffled other nonessential modes (see Methods). All three interventions significantly changed the mean firing rate of the network compared to the original models in the unperturbed, deafened, and perturbed singing cases (*p* < 10^−5^, two-sided Wilcoxon rank-sum test; Fig. S9A). To interpret the effects of intervention on the error landscape, we then tested the altered networks by changing both the degree of perturbation to auditory feedback (from correct tutor song patterns to completely random patterns; Fig. 5E) and to the input song (interpolating between songs of deafened birds and tutor song; Fig. 5F).

We found that disrupting the landscape modes strongly flattened the error landscape (Fig. 5E) and elevated error responses (Fig. S9), indicating that the landscape modes primarily alter the curvature of the error landscape. On the other hand, decorrelating the memory modes from tutor song moved the minimum location of the error landscape away from the tutor song and closer to silence (Fig. 5F), suggesting that the role of these modes is to shift the minimum of the error landscape. Paradoxically, disrupting the “other modes” could even slightly increased the slope of the error landscape and moved the minimum location closer to the original tutor song pattern (Fig. 5E-F). This suggests either that some memorization of the training data has occurred in these small modes (Bartlett et al., 2020) or that local learning has not fully converged at the fixed step size chosen in our experiments. These observations are qualitatively similar across alternative shuffling methods (Fig. S10).

Together, these results suggest that changes in the recurrent connectivity matrix of the E→ I→I model over the course of learning proceed in two phases: First, rapid learning of the landscape modes increases the gain of error responses and thereby sharpening the error landscape. Second, a relatively slower lock-in of memory modes aligns the minimum of the error landscape (smallest error response) with the tutor song pattern, providing a target for subsequent sensorimotor learning (Fig. 5G).

### 2.4 Model error codes suffice for reinforcement learning of song

In the previous sections, we have shown that our models, trained with local learning rules, produce sparse population error codes and that the learning process consists of an initial phase of error gain adjustment followed by a shift in minimum error response toward the tutor song. However, it remains to be seen whether these error codes contain sufficient information about deviations between vocal performance and the tutor song to guide learning. To test this, we developed a simple learning paradigm based on the actor-critic RL framework, widely used to model vocal learning in songbirds (R. Chen & Goldberg, 2020; R. Chen et al., 2019; Fiete et al., 2007; Kearney et al., 2019; Teşileanu et al., 2017; Tran & Koulakov, 2024).

**Figure 6:**
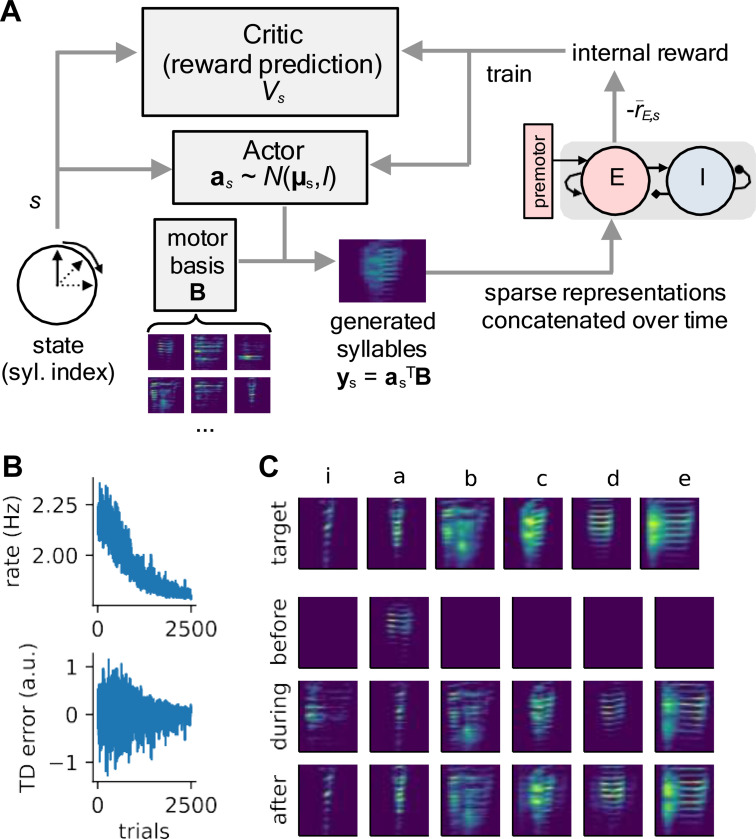
Error codes produced by local learning rules can be used to train a motor policy. **(A)** Schematic of the actor-critic reinforcement learning model. A syllable-based state variable s(t) indexes both the motor policy (actor a(s)) and the prediction of the expected reward (critic V(s)). Syllables y(s) are generated by linearly combining spectral basis elements **B** using the outputs of the policy a(s). The negative of mean error signal $-{\bar{r}}_{E,s}$ serves as the reward signal to be maximized. **(B)** Excitatory population firing rate (error signal) and temporal difference (TD) error plotted as a function of trial. Results shown are for the E→ I →E model, but results for the feedforward and premotor→E models are similar (see Fig. S11). **(C)** Tutor syllable templates (top row), and model-produced song before, during, and after learning (last three rows). The model is able to reproduce the tutor song using only the learned population error code as feedback.

We assume that the evaluation of song generation and learning happen at the syllable level. For every syllable index (“state”) s(t), the actor generates a set of weights a(s) used to linearly combine a set of spectrogram basis elements stored in the rows of a matrix B. That is, the predicted spectrogram is $a{\left(s\right)}^{⊤}B$ (Fig. 6A). As above, the generated spectrograms undergo a sparse linear embedding, forming the auditory population input to our local circuit model. Rather than maximizing external rewards from the environment, our agent is trained to minimize the population averaged error signal at each time step, equivalent to maximizing an internal reward signal equal to the negative of this quantity: $R(s)\propto -{\bar{r}}_{E,s}$, where ${\bar{r}}_{E}$ is the mean response of the excitatory population. The agent’s actions are parameterized as a state-dependent Gaussian distribution over basis elements: a(s)∼N(μ(s),I), and at each step of learning, the temporal difference (TD) error signal δ is used to update the agent’s estimate of the value of each state V(s). Specifically, after each syllable, these functions are updated by a standard temporal difference learning algorithm:

(1) $$\delta =-c{\bar{r}}_{E,s}-V(s)\;\text{(TD error)}$$

(2) μ(s)←μ(s)+αδ(a(s)−μ(s))

(3) V(s)←V(s)+αδ,

where α is the learning rate and c=10 is a constant scaling the mean rate.

Using this learning model, we tested the ability of all four of our models to copy a tutor song using their own error signals as a negative intrinsic reward. Learning in this highly simplified task converged quickly, within 2500 trials, as indicated by the exponential decrease in vocal error and the convergence of the TD error to zero (Fig. 6B; Fig. S11A,C). For the E→ E model, learning was slower, and only some generated syllables matched the target (Fig. S11D), likely because the population mean rates are largely insensitive to error (Fig. 3E). For the E → I→ E (Fig. 6C), as well as the premotor → E (Fig. S11A) and feedforward models, all the generated syllables gradually matched the target syllables. Furthermore, when landscape and memory connectivity modes were perturbed as in our analysis of the previous section, learning in these perturbed models was impaired, with perturbed memory modes having the strongest effect (Fig. S12). Together, these results demonstrate that the models’ learned error codes are sufficient to guide a motor program to produce songs that resemble a tutor target.

## 3 Discussion

For animals to learn complex behaviors without external reinforcement, they need an internal mechanism to evaluate their performance. Here, we have used computational models to explore the idea that this internal error is learned via local mechanisms orchestrating predictive cancellation of an expected sensory target. For any given sensory input, mismatches in this cancellation give rise to population error codes that can be used to move performance toward the target. Using songbird vocal learning as paradigmatic example, we implemented plausible circuit models with different connectivity motifs and types of plasticity that learn to cancel the auditory patterns of tutor songs during an early period of sensory learning. In the subsequent practice-based sensorimotor learning phase, auditory input to the system differs from the pattern of the tutor song, and an error code with similarities to observed responses in songbird auditory regions emerges. Among these models, the balanced excitation-inhibition network with Hebbian E → I and I → E plasticity rules (E → I → E model) best matched these experimental data. By characterizing changes to the weight matrix in the E → I → E model during learning, we also found that learning the cancellation proceeded in two steps: an initial sharpening of error responses, followed by a reshaping of the geometry of population errors that shifted minimum firing away from silence and toward the target pattern. Finally, we confirmed that the error codes generated by the E → I → E model are capable of guiding a motor policy to produce songs that match the target using a simple reinforcement learning model.

To our knowledge, this work offers the first concrete computational models of vocal error evaluation in a local forebrain circuit in songbird auditory areas. Given the rich connectivity among these auditory areas (Bolhuis & Moorman, 2015; Calabrese & Woolley, 2015) and the fact that several sites are likely to be important to sensory learning (Bolhuis & Moorman, 2015; Kearney et al., 2019; Mandelblat-Cerf et al., 2014; Roberts et al., 2017), we have treated them as a single balanced EI network model, assuming that the excitatory population both receives premotor information and sends error codes (Akutagawa & Konishi, 2010; Kearney et al., 2019; Mandelblat-Cerf et al., 2014; Nottebohm et al., 1982). Based on findings that tutor-song-selective neurons are present in area HVC during early sensory learning (Adret et al., 2012; Moseley et al., 2017; Nick & Konishi, 2005; Schroeder & Remage-Healey, 2024; Vallentin et al., 2016), we have also assumed that premotor input provides the temporal basis for predictive cancellation of tutor songs. While this idea of error codes arising from predictive cancellation is not new (e.g., Dempsey et al., 2019; Kennedy et al., 2014, in electric fish) and has been discussed in the context of novelty detection in the avian brain (Happ, 2023), ours is the first study to systematically examine both the implications of different types of plasticity in such a system and to demonstrate how the resulting error codes can subserve reinforcement learning.

Evidence from recent research offers some support for these results. Our model comparisons suggest that the E → I → E model is the most plausible (Fig. 4, Fig. S2), and studies have revealed age-contingent Hebbian plasticity from inhibitory interneurons to pyramidal neurons in mouse auditory cortex (D’amour & Froemke, 2015; Field et al., 2020; Vickers et al., 2018) that can affect both EI balance and sound frequency representation. Similarly, recent theoretical work shows that bi-phasic prediction error signals can emerge in EI networks through a combination of local learning rules in E → E and I → E connections (Asabuki et al., 2025). While the model in that work undergoes associative learning, our learning rules are anti-associative and produce an effective subtraction between sensory feedback and a learned predictive pattern. Encoding of prediction error patterns from such subtraction was also found in the single neuron responses in several auditory areas in anesthetized European starlings listening to conspecific songs (Rudraraju et al., 2024), consistent with a predictive coding model. Other support comes from the zebra finch brain, where the premotor area HVC projects sparsely to secondary auditory areas such as CM and Aiv via avalanche (Av) (Bolhuis & Moorman, 2015; Calabrese & Woolley, 2015), though these projections may not be dense enough (Akutagawa & Konishi, 2010; Mandelblat-Cerf et al., 2014; Roberts et al., 2017) to support the necessary learning in premotor → E models. However, the E → I → E model is robust to sparse premotor projections. In addition, spontaneous firing in Aiv (Mandelblat-Cerf et al., 2014) and CM (A. N. Chen & Meliza, 2018) may indicate low neuronal firing thresholds, to which our E→I→E is also robust.

Finally, to confirm that the learned error codes are sufficient to guide song generation, we implemented an actor-critic RL system whose components align with prominent theories about learning in the avian song system (R. Chen & Goldberg, 2020; Fee & Goldberg, 2011). For simplicity, we chose a scalar critic function and used the mean excitatory rates from our vocal error models to construct the temporal difference (TD) error, and under this assumption, the premotor→ E and E → I → E models are capable of learning to produce all syllables, while the E → E model results in matches between some syllables. Yet, our models potentially support multidimensional error signaling, as suggested by the correlation between the error codes and the true difference between the target and actual input patterns, (Fig. 3D), as well as the fact that anti-Hebbian learning encodes negative templates in the connections (Fig. S3).

Consistent with the vector-valued error code observed in our models, stimulation of ventral pallidum (VP) and Aiv, leading candidates for the critic and vocal error estimator, respectively, drives heterogeneous responses in VTA (R. Chen et al., 2019). These heterogeneous responses have recently been linked to vector-valued dopamine signals (Dabney et al., 2020; Gershman et al., 2024; Lee et al., 2024; Wärnberg & Kumar, 2023), for which several theories have been proposed. For example, heterogeneous dopamine responses have been suggested to encode prediction errors for different expectiles of the reward distribution (Dabney et al., 2020), TD errors corresponding to subspaces of cortical states (Lee et al., 2024), or gradients from the mismatch error between basal ganglia outputs and targets (Wärnberg & Kumar, 2023). An equally important question is how these vector dopamine signals are constituted physiologically. Here, we have proposed biologically plausible models that support such vector error codes, and a critical future step will be to connect these models to the existing theories of vector dopamine feedback.

Lastly, while we have attempted to align our models with known zebra finch physiology, we have made several key simplifications for purposes of theoretical tractability and computational efficiency: First, auditory inputs to our model are temporally interpolated sequences of sparse embeddings of song syllable spectrograms (Fig. S1) produced by a classic linear sparse coding model (Olshausen & Field, 1996, 1997). This is, at best, a caricature of early auditory processing and discards sub-syllabic variation. Even so, anti-Hebbian learning of the type we propose is theoretically agnostic to input distribution as long as these inputs maintain reasonable variability. Thus, our models could, in principle, handle sub-syllabic structure by a proper choice of time constants and input scaling. Second, our models learn through bilinear Hebbian or anti-Hebbian rules with fixed thresholds (Fig. 2C and Eq. 7). In reality, the plasticity can be bounded, adaptive, and multi-factored. In this work, we have elided these complexities, focusing simply on what is learnable in principle by simple local rules. Third, we have focused on firing rates and rate-based neurons. Future studies will need to extend these results to the added sparsity and nonlinearities of spiking neural networks. Fourth, we do not presume to account for all auditory processing. While responses of our proposed cancellation circuit are minimal when the bird’s own song closely matches the tutor memory, we do not propose that the bird fails to perceive its own song in these cases. That is, the songbird’s predicament differs from other situations in which the goal of corollary discharge is to *perceptually* cancel predictable nuisance inputs (Kennedy et al., 2014; Schneider et al., 2014, 2018).

In summary, our models suggest that local learning for predictive cancellation is sufficient for the formation of error signals that can guide self-driven learning of complex motor behaviors. Further, by comparing models with different types of plasticity and aligning these to experimental data, we predict that a circuit involving plasticity between excitatory and local inhibitory neurons is most likely to support learning of internal behavioral evaluation signals. Together, these results constitute a novel computational theory for the circuit mechanisms underlying animals’ ability to evaluate and refine their behaviors without external supervision or reinforcement.

## 4 Method

### 4.1 Vocal error circuit models

The vocal error circuit models can be described by a set of differential equations for the total input $h^{a}\in {\mathbb{R}}^{N_{a}}$ (where a=E,I)

(4) $$\tau_{E}\frac{\text{d}}{\text{d}t}h^{E}=-h^{E}+W_{E}r^{H}+J^{EE}r^{E}-J^{EI}r^{I}+y+\epsilon_{t}^{E}$$

(5) $$\tau_{I}\frac{\text{d}}{\text{d}t}h^{I}=-h^{I}+J^{IE}r^{E}-J^{II}r^{I}+\epsilon_{t}^{I}$$

where $r^{E}=\phi_{E}\left(h^{E}\right)$, $r^{I}=\phi_{E}\left(h^{I}\right)$ and $r^{H}\in {\mathbb{R}}^{N_{H}}$ denote the excitatory, inhibitory, and premotor neuron firing rates, respectively, the τ’s are time constants, and

$$\phi_{b}(x)=\frac{r_{max}}{2}\left[1+\text{erf}\left(\frac{x-\theta_{b}}{s_{b}\sqrt{2}}\right)\right]$$

are sigmoidal activation functions. Values of the parameters for the main results are summarized in Table 1. Reasonable choices of maximum firing rates $\left(r_{max}^{I}\geq r_{max}^{E}\right)$ did not qualitatively affect the dynamics. $W_{E}$ are the synaptic weights from premotor input to the excitatory neurons, $J^{ab}\geq 0$ are local synaptic connections from population b to population a, y is the auditory input, and $\epsilon_{t}^{a}∼N\left(0,\sigma_{\epsilon }\right)$ is the i.i.d. white noise at each time step.

#### 4.1.1 Connectivity Matrices

As noted above, the synaptic connections are represented by a set of weight matrices: $W_{E}$ denotes premotor→E connections and $J^{ab}\geq 0$ are connections from population b to population a.

The values of the *initial* weights of existing synapses in the premotor→ E connections are drawn from a log-normal distribution:

$${\left.W_{ij}\right|}_{c_{ij}^{W}=1}∼\text{LogNorm}\left(\mu_{W0},\sigma_{W0}^{2}\right).$$

where $c_{ij}^{W}\in \{0,1\}$ is a Bernoulli random variable with probability $P\left(c_{ij}^{W}\right)={\bar{c}}_{W}$ and denotes whether the synapse from $r_{j}^{H}$ to $r_{i}^{E}$ exists. In the E→E and E→I→E models, $W_{ij}$ stays fixed, while in the feedforward and EI networks with premotor→E plasticity, it undergoes learning.

For the feedforward model, $J^{ab}=0$, which reduces the equations to

(6) $$\tau_{E}\frac{\text{d}}{\text{d}t}r^{E}=-r^{E}+\phi_{E}\left(W_{E}r^{H}+y\right).$$

For the EI network models, the *initial* weights obey

$${\left.J_{ij}^{ab}\right|}_{c_{ij}^{Jab}=1}∼\text{LogNorm}\left(\frac{J_{0}^{ab}}{\sqrt{N_{b}}},\frac{{\left(\gamma_{ab}J_{0}^{ab}\right)}^{2}}{N_{b}}\right),$$

where $c_{ij}^{Jab}\in \{0,1\}$ is again a Bernoulli random variable denoting whether the synapse from $r_{j}^{b}$ to $r_{i}^{a}$ exists, $J_{0}^{ab}$ controls the scales of the connections, and $\gamma_{ab}$ controls the variances. Other choices of $\gamma_{ab}$ with similar order of magnitude gave qualitatively similar results. For simplicity, we assume all recurrent weights have the same density, i.e. $P\left(c_{ij}^{JEE}\right)=P\left(c_{ij}^{JIE}\right)=P\left(c_{ij}^{JEI}\right)=P\left(c_{ij}^{JII}\right)={\bar{c}}_{J}$.

To enforce EI balance, the $J_{0}^{ab}$ obey the EI balance condition

$$\frac{J_{0}^{EE}}{J_{0}^{IE}}<\frac{J_{0}^{EI}}{J_{0}^{II}}.$$

One can easily derived this inequality by noticing that the mean recurrent input to each neuron is $O(\sqrt{N\bar{c}})$ and must be set to zero:

$$\frac{I_{ext}}{\sqrt{N_{E}{\bar{c}}_{J}}}+J_{0}^{EE}{\bar{r}}_{E}-\sqrt{\frac{N_{I}}{N_{E}}}J_{0}^{EI}{\bar{r}}_{I}\approx O\left(\frac{1}{\sqrt{N_{E}{\bar{c}}_{J}}}\right)\approx 0\;\text{excitatory neuron input}$$

$$J_{0}^{IE}{\bar{r}}_{E}-\sqrt{\frac{N_{I}}{N_{E}}}J_{0}^{II}{\bar{r}}_{I}\approx O\left(\frac{1}{\sqrt{N_{E}{\bar{c}}_{J}}}\right)\approx 0\;\text{inhibitory neuron input,}$$

where $I_{ext}$ is the total external input to each excitatory neuron and is assumed to be as strong as $O\left(\sqrt{N_{E}}\right)$. Note that, in contrast with van Vreeswijk and Sompolinsky (1998), the inhibitory population in our models does not receive strong external input. Unless otherwise mentioned, Table 1 summarizes the values of the parameters. Other values obeying the balance condition did not change the qualitative results.

For simplicity, $c_{ij}^{W}$ and $c_{ij}^{Jab}$ are fixed once the networks are initialized. That is, there is no birth or death of synapses during the simulations. Except for Fig. 3C (left), $P\left(c_{ij}^{W}\right)=1$ for the feedforward and premotor→ E models, and for E → E and E → I → E models, $P\left(c_{ij}^{W}\right)=0.05$ for learning realistic input. In Fig. 3C (left), $P\left(c_{ij}^{W}\right)$ was varied from 0 to 1 to see its effect on the training in different models. Unless otherwise specified, ${\bar{c}}_{J}=0.5$, though denser (up to 1) or sparser (0.3) recurrent connectivity did not show qualitatively different results.

### 4.2 Hebbian and anti-Hebbian learning

In the premotor→ E and E → E models, anti-Hebbian learning happens in either premotor→ E or E → E connections. In the E → I→ E model, Hebbian learning happens in E → I and I → E connections. Both learning rules can be described by the same differential equation, where the learning strength η is strictly positive for Hebbian learning, and strictly negative for anti-Hebbian learning.

(7) $$\tau_{W}\frac{\text{d}}{\text{d}t}W_{ij}=-\left(W_{ij}-w_{0}\right)+\eta \left[r_{i}(t)-\theta_{\text{active}}^{i}\right]\left[r_{j}\left(t-{\text{Δ}}_{t}\right)-\theta_{\text{active}}^{j}\right],$$

where $W_{ij}$ is the synaptic weight from neuron j to neuron i, $\tau_{W}\gg 0$ is the time constant of the weight evolution, $w_{0}$ is the weight baseline, ${\text{Δ}}_{t}$ is the time asymmetry of the plasticity, and $\theta_{\text{active}}∼O(1)$ are thresholds for determining whether the pre- or postsynaptic neurons are active. The first term is used to prevent the weights from growing to infinity. The second term learns the correlation or anti-correlation between the pre- and postsynaptic neurons. Briefly, in (anti-)Hebbian learning, the synaptic weight is increased (decreased) when both the pre- and postsynaptic neurons are active, and decreased (increased) when they are not co-active. To obey Dale’s law, the weights are clipped at each time step:

$$W_{ij}\leftarrow \max\left\{W_{ij},0\right\}.$$

Values of the parameters are summarized in Table 1.

### 4.3 Premotor Input

We assume that premotor neurons in our model respond to tutor song, as has been found for HVC (Adret et al., 2012; Moseley et al., 2017; Nick & Konishi, 2005; Schroeder & Remage-Healey, 2024; Vallentin et al., 2016). Specifically, the firing rate of a premotor neuron $r_{i}^{H}$ for $i=1,2,\ldots ,N_{H}$ in multiple renditions $j=1,2,\ldots ,N_{\text{rend}}$ is modeled as

(8) $$r_{i}^{H}(t)=\sum_{j}r_{\text{burst}}^{\left(j\right)}\exp\left[\frac{1}{2{\left(\tau_{\text{burst}}^{\left(j\right)}\right)}^{2}}{\left(t-t_{on}^{\left(j\right)}-\frac{T_{\text{song}}}{N_{H}-1}(i-1)-\delta_{\text{burst}}^{\left(j\right)}\right)}^{2}\right]$$

where $r_{\text{burst}}^{\left(j\right)}$ is the burst firing rate, $\tau_{\text{burst}}^{\left(j\right)}$ is the peak width, $t_{on}^{\left(j\right)}$ is the song onset, $T_{\text{song}}$ is the song length, and $\delta_{\text{burst}}^{\left(j\right)}$ is the jittering for the burst peak. Notice that $r_{\text{burst}}^{\left(j\right)}$, $\tau_{\text{burst}}^{\left(j\right)}$, and $\delta_{\text{burst}}^{\left(j\right)}$ can vary over renditions.

Except for Fig. S2, we further assume that premotor neurons have already developed sparse bursting behaviors with stable burst times and relatively stable burst profiles during a rendition of song. We set $r_{\text{burst}}^{\left(j\right)}∼N(150,0.1)\;\text{Hz}$, $\tau_{\text{burst}}^{\left(j\right)}∼N(20,0.01)\;\text{ms}$, and $\delta_{\text{burst}}^{\left(j\right)}=0\;\text{ms}$.

We also tested the models using developing, progressively regular, premotor bursting profiles in Fig. S2. In these cases, $r_{\text{burst}}^{\left(j\right)}$ follows a log-normal distribution with mean 150 Hz and standard deviation 70ψ(j)Hz, $\left(\tau_{\text{burst}}^{\left(j\right)}-20\right)$ follows an exponential distribution with scale parameter 60ψ(j)ms, and $\delta_{\text{burst}}^{\left(j\right)}∼N(0,100\psi (j))\;\text{ms}$, where

$$\psi (j)=\exp\left(-3j/N_{\text{rend}}\right)$$

is a scaling factor decreasing smoothly over training.

### 4.4 Auditory Input

Except for Fig.1A, we tested the models using input patterns derived from birdsong data. To simulate input to the secondary auditory regions, we postulate that the upstream auditory processing implements a form of sparse coding (Lewicki & Sejnowski, 2000; Olshausen & Field, 1997, 2004) (see 4.5). We trained a sparse coding model on flattened spectrograms of tutor song, similar to what a juvenile bird might hear during the sensory phase. More specifically, the training data involved the syllables of adult zebra finches, vocalizations of juvenile zebra finches (35–40 dph), and behavioral noise recorded during 35–40 dph. After training, the sparse coding model trained using test sequences of syllable spectrograms, generating sequences of discrete syllable representations. To produce time series whose durations matched the original song recordings, each representation was extended over the time window of the corresponding syllable, with the gaps between filled by zeros. Lastly, the time series were convolved with an exponential kernel to smooth the transitions between representations.

#### 4.4.1 Perturbation and Deafening

To simulate external perturbation of auditory feedback (except for Fig.5F), we first added 100ms of white noise to the audio clips of a particular syllable, generating the spectrograms using the same parameters as for unperturbed song. The spectrograms of both perturbed and unperturbed syllables are then fed into the sparse coding model in the same syllable order, and the continuous time series were generated following the same procedures as for the unperturbed input (Fig.S1). To simulate the deafening case, we set the auditory input y=0.

To quantify the changes of error landscape in Fig. 5F, we directly perturbed the sparse embedding of the tutor song input because the alternative perturbation approach described above leads to great variance in the effective difference between perturbed and correct tutor song patterns for each choice of perturbation strength (Fig. S1G). First, a noise pattern, $\tilde{y}$ is generated by randomly shuffling the elements of the sparse embedding of the syllable to be perturbed. Given a perturbation strength variable γ, the new perturbed pattern, $\hat{y}(\gamma )$ was calculated as

$$\hat{y}(\gamma )=\sqrt{1-\gamma^{2}}y+\gamma \tilde{y}.$$

For Fig. 5G, we simply scaled the sparse embedding by a scaling parameter $a:\hat{y}(a)=ay$, where a=0 corresponds to the deafening case and a=1 corresponds to the correct singing case.

#### 4.4.2 Simplified input for illustrating error landscape

The error landscape at a given moment in time is the transient neuronal activation around that moment. To be able to clearly visualize an error landscape in Fig.1A, we used a simplified auditory input pattern $\xi \in {\mathbb{R}}^{N_{E}}$ with each element drawn from an i.i.d. Gaussian distribution, $\xi_{i}∼N\left(0,I_{3\times 3}\right)$, though the results do not depend on the mean and standard deviation as long as they are O(1). The premotor input is 1-dimensional. We would like to focus on a particular moment in time and thus set $r_{H}(t)=r_{\max}^{H}$ and y(t)=ξ. We then used the stationary population mean rates of the excitatory neurons at different auditory patterns to plot the error landscape.

### 4.5 Sparse Coding

Sparse coding theory postulates that neurons form an overcomplete set of basic sensory features, with only a small subset of neurons are activated for a particular sensory input (Lewicki, 2002; Lewicki & Sejnowski, 2000; Olshausen & Field, 1996, 1997, 2004). Variants of sparse coding models have predicted key properties of sensory encoding (Beyeler et al., 2019; Mukherjee et al., 2025; Olshausen & Field, 2004), including in primary visual cortex (Olshausen & Field, 1996, 1997), primary and secondary auditory regions (Lewicki, 2002; Mukherjee et al., 2025), and, pertinently to our work, in the caudo-medial nidopallium (NCM) of European starling (Kozlov & Gentner, 2016), an auditory region containing neurons selectively tuned to birdsong.

We adapted a classic linear sparse coding model (Olshausen & Field, 1996, 1997). Briefly, let $X\in {\mathbb{R}}^{K\times N}$ be a set of flattened spectrograms of bird song syllables, where K is the number of samples and N is the number of time bins multiplied by the number of frequency bins. We would like to find basis features $A\in {\mathbb{R}}^{L\times N}$, where L is the number of features, and sparse coefficients (i.e. the neural responses) $S\in {\mathbb{R}}^{K\times L}$, such that

X=SA+ϵ

where ϵ is some noise. In the original work(Olshausen & Field, 1997), this was formulated as a probabilistic inference problem and approximated such that its solution for a training set $\tilde{X}$ can be found by repeating the following three steps until convergence of the basis $A^{*}$:

1. For a fixed A, find
$$S^{*}=\arg\;\underset{S}{\min}\frac{1}{N}\sum_{ij}{\left({\tilde{X}}_{ij}-{\left(SA\right)}_{ij}\right)}^{2}+\frac{\lambda }{L}\sum_{ik}\left|S_{ik}\right|$$
2. For the optimized $S^{*}$, find
$${\hat{A}}^{*}=\arg\;\underset{A}{\min}\sum_{ij}{\left({\tilde{X}}_{ij}-{\left(S^{*}A\right)}_{ij}\right)}^{2}$$
3. Normalize each basis, $A_{ij}^{*}={\hat{A}}_{ij}^{*}/\sigma_{Aj}$, where $\sigma_{Aj}$ is the standard deviation of the j-th column vector.

We performed this optimization for a set $\tilde{X}$ comprising flattened spectrograms of normal syllables from adult male zebra finch songs (Koch, 2024), immature vocalization (Brudner et al., 2023), and behavioral noise. The model targeted L=100 basis elements, a highly overcomplete set for song, which is effectively low-dimensional at the syllable level (d<10; Fig. S1A; Goffinet et al., 2021). Using the learned basis $A^{*}$, we then repeated Step 1 to find the sparse responses $S^{*}$ for normal syllables and syllables overlapped with 100-ms white noise, which were then used to train the vocal error model.

To map the L-dimensional sparse representations to $N_{E}>L$ excitatory neurons, we tried neighbor and random mappings, which resulted in qualitatively similar dynamics. For neighbor mapping, the i-th element of an L-dimensional sparse representation is the input to the excitatory neurons with indices from $(i-1)\times ⎿N_{E}/L⏌+1$ to $i\times ⎿N_{E}/L⏌$. For random mapping, we generated a projection matrix $M\in {\mathbb{R}}^{N_{E}\times L}$ with elements each drawn i.i.d. from a unit normal distribution. While we found both approaches resulted in similar dynamics, the random mapping approach resulted in inputs that were more Gaussian and less sparse. Thus, to preserve input sparsity, we used neighbor mapping for this work.

### 4.6 Experimental Methods

#### 4.6.1 Surgery

Adult male birds were anesthetized with inhaled 1.5–2% isofluorane and placed on a head stereotax with a temperature-regulated bed. The incision was sterilized with alcohol, and a local anesthetic (bupivacaine 0.25%) was applied. Coordinates for the injections and lens implant were measured from the bifurcation of the midsagittal sinus. The coordinates used for targeting various auditory regions were as follows: CM, 43, 1.8A, 1.95L, 0.7 and 0.9V, 2.0A, 2.05L 0.65 and 0.95V, 2.2A, 1.95L 0.7 and 0.9V. Birds were injected with 200nL of an AAV2/9 CAG GCaMP6s (Addgene, 100844-AAV9) viral construct on each site at a speed of 9nl/s using a Nanoject-II (Drummond Scientific). Immediately after the injections, we lowered a GRIN prism lens (Inscopix 1050–004601) held by a vacuum holder at a low speed (10um/s) centered around the injection sites and facing medially. After lowering, the lens was cemented to the skull and covered using body-double to prevent damage. Three weeks after the lens implantation we examined the field of view and cemented a baseplate to attach a 1-photon miniature microscope (nVista 3.0, Inscopix. After two days of surgery recovery, the bird was attached to the microscope using a counterweight system to ensure normal behavior.

#### 4.6.2 Deafening

Birds were deafened by bilaterally removing the cochlea. Birds were anesthetized with 20 mg/kg of ketamine and 10mg/kg xylazine and fixed on their side on a movable platform to facilitate access to the ear. The incision was sterilized with alcohol and anesthetized with bupivacaine 0.25%. The ear skin was cut using microscissors, and the tympanic membrane was punctured by a fine scalp. Then, the columella and the footplate were detached with forceps to reveal the oval window. A fine custom tungsten wire hook was introduced into the inner ear canal to remove the cochlea entirely. After surgery recovery (as measured by normal singing rates), the birds were re-attached to the miniscope to image the neurons after deafening.

#### 4.6.3 Calcium imaging

Imaging data was acquired with a nVista 3.0 data acquisition board and the Inscopix imaging software at a frame rate of 12 Hz, using an LED power of 1–1.4mW/mm2. The acquisition board interfaced with custom Matlab code to monitor singing. The computer detected changes in amplitude when the bird started to sing to trigger the imaging system for sessions of 1 minute and record the audio data. The imaging files recorded within a day were curated by concatenating all the sessions that contained singing or playbacks together, spatially downsampling the videos, performing motion correction, and extracting the ROIs using CNMFe (Zhou et al., 2018). The criteria for non-inclusion comprised sessions that had dropped frames or sessions that were accidentally triggered and did not contain song. The audio recordings containing song were manually labeled to determine song onsets and offsets. To avoid contamination of the song signal due to the low calcium sensor decay, only the songs that had at least 2 seconds of silence were labeled. Then, the extracted traces were aligned to the onset time stamps, z-scored, and averaged across trials. To generate the scatter plots, the activity was averaged over the first 2 seconds of song.

#### 4.6.4 Cell registration

Cell registration pre- and post-deafening was carried out using CellReg (Sheintuch et al., 2017). This algorithm used the spatial footprint matrices of the fields of view generated by the neuron extraction performed previously (Zhou et al., 2018) to obtain a matrix of indices that corresponded to the same neurons across the sessions.

### 4.7 Model Metrics

#### 4.7.1 Percentage of Active Excitatory Neurons

To measure the sparseness of the population responses, we calculated the percentage of active excitatory neurons at every time step and then averaged over time window $\left[t_{0},t_{1}\right]$, given by

$$P_{\text{active}}=\frac{1}{t_{1}-t_{0}}\frac{1}{N_{E}}\sum_{t_{0}<t<t_{1}}\sum_{i=1}^{N_{E}}H\left(r_{i}^{E}(t)-\theta_{\text{active}}\right)$$

where H(x)=1 if x≥0 and H(x)=0 otherwise, and $\theta_{active}=5\;\text{Hz}$ is the threshold chosen close to the spontaneous mean rate. Other values such as 3 Hz produced qualitatively similar results.

#### 4.7.2 Cosine Similarity

We used cosine similarity to measure the relationship between the firing patterns across the neuron population and the auditory input patterns. For a population of N neurons, the cosine similarity $S_{c}$ between the population vector at time t, $r(t)={\left[r_{1}(t),r_{2}(t),\ldots ,r_{N}(t)\right]}^{T}$, and auditory pattern (either stationary or at some time step) $x={\left[x_{1},x_{2},\ldots ,x_{N}\right]}^{T}$ is

$$S_{c}(r(t),x)=\frac{x^{T}r(t)}{\|x{\|}_{2}\|r(t){\|}_{2}},$$

where $\|\cdot {\|}_{2}$ denotes the Euclidean norm.

#### 4.7.3 Quality of Cancellation

To measure the quality of cancellation, we compared how strongly the neurons represent the tutor song during correct singing trials before and after training. The strength of representation is measured by the cosine similarity described above. For each simulation, the quality of cancellation, averaged over the singing time window from song onset $t_{0}$ to song offset $t_{1}$, is then

$$Q_{\text{cancel}}=\frac{1}{t_{1}-t_{0}}\sum_{t_{0}<t<t_{1}}\left[S_{c}\left(r_{\text{untrained}}(t),y_{TS}\right)-S_{c}\left(r_{\text{trained}}(t),y_{TS}\right)\right]$$

where the superscript (i) denotes the i-th trial. The larger this quantity, the more effectively the cosine similarity between neural firing and the tutor song pattern is reduced. Negative values indicate that, on average, the representation of the tutor song is enhanced after training.

#### 4.7.4 Normalized Neuronal Activity

Figs. 4, S2, S4, S6, and S7 contain normalized neuronal activity. For experimental data, the raw trial-averaged calcium activity of each ROI was first subtracted by the baseline (activity averaged over the 1-second window before singing onset). For Fig. 4B, the baseline-subtracted calcium activity was averaged over the 2-second time window after singing onset. We chose a longer window than song duration (~1s) because of the low sampling rate (12Hz) and low-pass filtering effect of one-photon calcium imaging. Finally, for visualization purpose in Fig. 4B, all the averaged activities were divided by the standard deviation across ROIs in the correct singing case (i.e., the x and y axes are rescaled by a common factor).

For Figs. 4C, S2D, S4B, S6D, and S7B, the processing is similar—trial averages subtracted by the baseline and averaged over singing time window—except that the activity was averaged over the song duration (~1s) after singing onset instead of a 2-second time window. To get the trial averages, each trained model is simulated for 20 different initial conditions for Figs. 4, S4, S6, and S7, and for 50 different initial conditions for Fig. S2.

### 4.8 Bidirectional Sorting of Neuronal Activity

To visualize the sequential activation and sequential inhibition in Fig. S7, we developed a two-step process: First, the neurons were grouped into either excited or inhibited after song onset by comparing the mean activities before (500 ms for calcium imaging data and 100 ms for models) and after song onset (1500 ms for calcium imaging data and 1000 ms for models). Then, the excited group was sorted by the latency to the peak (maximum standardized activity) of each neuron, and the inhibited group was sorted by the latency to the dip (minimum standardized activity) of each neuron.

### 4.9 Connectivity Mode Analysis

To analyze the recurrent dynamics, we performed singular value decomposition (Fig. 5A) on the recurrent weight matrix over training time t,

(9) $$J_{t}=\left(\begin{array}{cc} J_{t}^{EE} & -\left|J_{t}^{EI}\right|\\ J_{t}^{IE} & -\left|J^{II}\right| \end{array}\right)=U_{t}\mathrm{diag}\left(s_{t}\right)V_{t}^{T},$$

where the columns of U and V are the left and right singular vectors, respectively, and s contains the corresponding singular values. Notice that we pull out the negative sign of the inhibitory weights explicitly to avoid confusion. The left singular vectors ${\text{U}}_{i}$ (“modes”) define the orthogonal directions to which the dynamics maps the population vector $h={\left[h_{1}^{E},\ldots ,h_{N_{E}}^{E},h_{1}^{I},\ldots ,h_{N_{I}}^{I}\right]}^{T}$, where $h_{i}^{a}$ is the total input to the i-th neuron of population a. To see this, consider $\tau_{E}\approx \tau_{I}=\tau$ and rewrite Eqs. 4–5 as

(10) $$\tau \frac{\text{d}}{\text{d}t}h=-h+\mathrm{Jr}+\epsilon =-h+U\left(\mathrm{diag}(s)V^{T}r\right)+\epsilon \equiv -h+\sum_{i}l_{i}U_{i}+\epsilon ,$$

where h is a concatenation of $h^{E}$ and $h^{I}$ and similarly for r, and $l_{i}$ is the i-th element of $\text{diag}(s)V^{T}r$.

#### 4.9.1 Dissimilarity and Memory Encoding

The dissimilarity for each mode i at the n-th rendition is defined as the maximum cosine similarity between itself and *any* mode at time 0:

$$\underset{j}{\max}\left|v_{i}^{T}(n)v_{j}(0)\right|,$$

where v is the corresponding left singular vector, and the 0-th rendition is the time prior to learning.

Similarly, to quantify the memory encoding for each mode i, we calculated

$$\underset{k}{\max}\left|\text{Corr}\left(v_{i},y_{TS,k}\right)\right|,$$

the maximum correlation between that mode and $y_{TS,k}$ the discrete pattern corresponding to the k-th syllable of the tutor song.

#### 4.9.2 Convergence Time

We defined convergence time $\tau_{c}$ for our models as

$$\tau_{c}=\min\left\{t\left|\frac{x_{t}-x_{\min}}{x_{\max}-x_{\min}}>\theta_{\tau c}\right.\right\},$$

where $x_{\min}$ and $x_{\max}$ are the minimum and maximum of ${\left\{x_{t}\right\}}_{t}$, the parameter of the learning curve, and $\theta_{\tau c}$ is a threshold. We chose $\theta_{\tau c}=0.8$. For learning in our data t is measured in trials, such that δt=1. In our analysis of network modes, we measured the convergence time for mean curves of the landscape and memory modes.

#### 4.9.3 Perturbing the Modes

To probe the roles of different connectivity modes on the error codes represented by the excitatory neurons, we disrupted the excitatory patterns of the modes in several ways. For non-memory modes, i.e., those not correlated with the tutor song patterns, we directly perturbed their patterns using the following approaches:

1. shuffling all elements of the selected left singular vectors;
2. shuffling the first $N_{E}$ elements of the selected left singular vectors;
3. replacing the selected left singular vectors with patterns drawn from a zero-mean i.i.d. Gaussian distribution;
4. replacing the selected left singular vectors with zeros;
5. swapping the selected left singular vectors with the least significant left singular vectors.

All approaches produced qualitatively similar results. The first approach was used in Fig. 5, while the results from the others are shown in Fig. S10. Each perturbed left singular vector is scaled to have Euclidean norm equal to 1, except for Approach 4, which replaces the vectors with zeros.

For memory modes, we “de-memorized” them by subtracting the memory components from the left singular vectors. More specifically, denoting the excitatory part of the left singular vectors by $U_{E}\in {\mathbb{R}}^{K\times N_{E}}$, where K is the number of memory modes, the matrix of “de-memorized” vectors ${\hat{U}}_{E}$ is

$${\hat{U}}_{E}=U_{E}-U_{E}M^{T}M$$

where the rows of $M\in {\mathbb{R}}^{L\times N_{E}}$ are a set of orthonormal basis for L syllable patterns $\left(MM^{T}=\right.\left.I_{L\times L}\neq M^{T}M\right)$. The “de-memorized” row vectors in ${\hat{U}}_{E}$ are orthogonal to the basis vectors for the syllable patterns:

$${\hat{U}}_{E}M^{T}=U_{E}M^{T}-U_{E}M^{T}MM^{T}=U_{E}M^{T}-U_{E}M^{T}I=0.$$

Finally, each row of ${\hat{U}}_{E}$ is scaled to have Euclidean norm equal to 1.

## Supplementary Material

Supplement 1

## Figures and Tables

**Table 1:** Summary of comparisons between models and experiments. The base case corresponds to simulations with regular premotor firing during training, premotor projection density favored by different models (dense for the feedforward and premotor →E models, and sparse for the E→E and E→I→E models; see Table 1), and whole-syllable perturbations (changing the input patterns for the entire duration of the syllable). The other conditions deviate from the base case as described.

Test	Auditory feedback during singing	Feedforward	Premotor→E	E→E	E→I→E
Base case	perturbed deafened	✓ ×	✓ ✓	✓ ×	✓ ✓
Irregular premotor firing during training	perturbed deafened	× ×	✓ ×	✓ ×	✓ ✓
Ultra-sparse premotor projections (5%)	perturbed deafened	× ×	× ×	✓ ×	✓ ✓
Sub-syllabic perturbation	perturbed	×	✓	×	✓

## Footnotes

† Note that Hebbian plasticity in the I → E connections is *effectively* anti-Hebbian, since the I → E connections are inhibitory.

* Certain memory modes can also have an effect on the shape of the error landscape. To isolate the effect of the components correlated with the tutor song patterns, we did not directly shuffle their entries.

## 4.10 Code Availability

The code for simulating the models, running the analyses, and generating the figures in this work can be found in https://github.com/gongziyida/vocal-error-network.git.
