# Supplementary material for: Correctness is its own reward: bootstrapping error signals in self-guided reinforcement learning
Source: bioRxiv. 2025 Aug 19:2025.07.18.665446. Preprint. [Version 2] doi: 10.1101/2025.07.18.665446 (PMC12393258; doi:10.1101/2025.07.18.665446)
Supplement: Supplement 1 [file media-1.pdf]

Correctness is its own reward: bootstrapping error signals in self-guided reinforcement learning

Ziyi Gong[1], Fabiola Duarte[1] , Richard Mooney[1,2], John Pearson[1,3]

[1]Department of Neurobiology, Duke University, Durham, NC, USA
[2]Department of Cell Biology, Duke University, Durham, NC, USA
[3]Department of Electrical and Computer Engineering, Duke University, Durham, NC, USA

Supplementary Figures and Parameter Tables

Symbol	Value	Definition
N_E	600	Number of exc. neurons
N_I	150	Number of inh. neurons
N_H	15	Number of premotor neurons
τ_E	30	Exc. neuron time constant
τ_I	10	Inh. neuron time constant
σ_ϵ	0.1	Neuronal noise standard deviation
$r^E_{\max}$	100	Max. exc. neuron firing rate
$r^I_{\max}$	100	Max. inh. neuron firing rate
θ_E	6	Exc. neuron firing threshold (premotor→E / feedforward model)
θ_E	0	Exc. neuron firing threshold (E→I→E / E→E model)
θ_I	0	Inh. neuron firing threshold
s_ϕ	2	Neuronal activation function gain
$\bar{r}_{\text{burst}}$	150	Mean peak firing rate of premotor burst
$\bar{\tau}_{\text{burst}}$	20	Mean peak width of premotor burst
$\bar{\delta}_{\text{burst}}$	0	Mean jittering of premotor burst
$\bar{c}_J$	0.5	Recurrent connectivity probability
J^{EE}_0	0.1	Scale parameter for E→E connections, $\mathbf{J}_{EE}$
J^{EI}_0	0.17	Scale parameter for I→E connections, $\mathbf{J}_{EI}$
J^{IE}_0	0.1	Scale parameter for E→I connections, $\mathbf{J}_{IE}$
J^{II}_0	0.15	Scale parameter for I→I connections, $\mathbf{J}_{II}$
γ	0.1	Recurrent connectivity variance parameter
μ_{W0}	1/15	Premotor projection mean
$\bar{c}_W$	1	Premotor→E connectivity probability (premotor→E / feedforward model)
$\bar{c}_W$	0.05	Premotor→E connectivity probability (E→I→E / E→E model)
τ_W	10000	Synaptic plasticity time constant
θ^E_{active}	1.5	Plasticity threshold for exc. neurons
θ^I_{active}	5	Plasticity threshold for inh. neurons
θ^H_{active}	0	Plasticity threshold for premotor neurons
Δt_H	10	Plasticity time asymmetry (post - pre) for premotor→E synapses
Δt_{EE}	10	Plasticity time asymmetry (post - pre) for E→E synapses
Δt_{IE}	10	Plasticity time asymmetry (post - pre) for E→I synapses
Δt_{EI}	0	Plasticity time asymmetry (post - pre) for I→E synapses
η_H	-0.03	Learning strength for premotor→E synapses (premotor→E model)
η_{EE}	-0.05	Learning strength for E→E synapses (E→E model)
η_{EI}	0.05	Learning strength for I→E synapses (E→I→E model)
η_{IE}	0.006	Learning strength for E→I synapses (E→I→E model)

Table 1: **Model parameters in the main results, unless otherwise specified.**

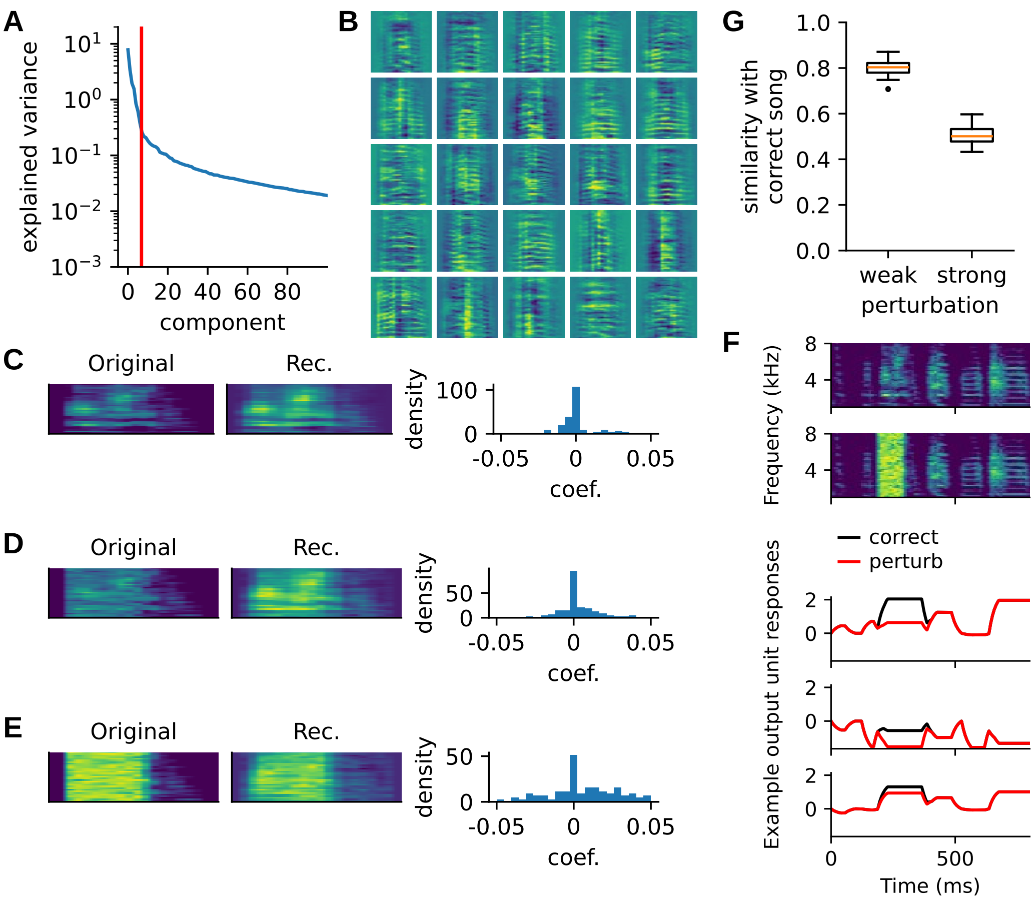

Figure S1: **Sparse coding of auditory input.** **(A)** The effective dimension (red) of tutor song syllable spectrograms, given by the participation ratio d from the eigenvalues λ_i of the covariance matrix between the flattened spectrograms. The participation ratio is given by $d = (\sum_i \lambda_i)^2/(\sum_i \lambda_i^2)$. Intuitively, if there are K large eigenvalues $\lambda_1 \approx \lambda_2 \approx ... \approx \lambda_K \approx \bar{\lambda}$, $d \approx K^2\bar{\lambda}^2/(K\bar{\lambda}^2) = K$ (Gao et al., 2017). **(B)** Examples of learned basis elements. **(C-E)** Examples of reconstruction using the bases and corresponding coefficients (i.e., the responses of the sparse coding model; right column) for a normal syllable (C), weakly perturbed syllable (D), and strongly perturbed syllable (E). Right: histograms showing the distribution of coefficients for each reconstruction. **(G)** Responses from the sparse coding model for the weakly perturbed syllables are more similar to the correct syllable than those for the strongly perturbed syllables. **(F)** Syllable-specific neural responses over time. Top two rows show an example of correct song and perturbed song. Bottom three rows show three example auditory coding units.

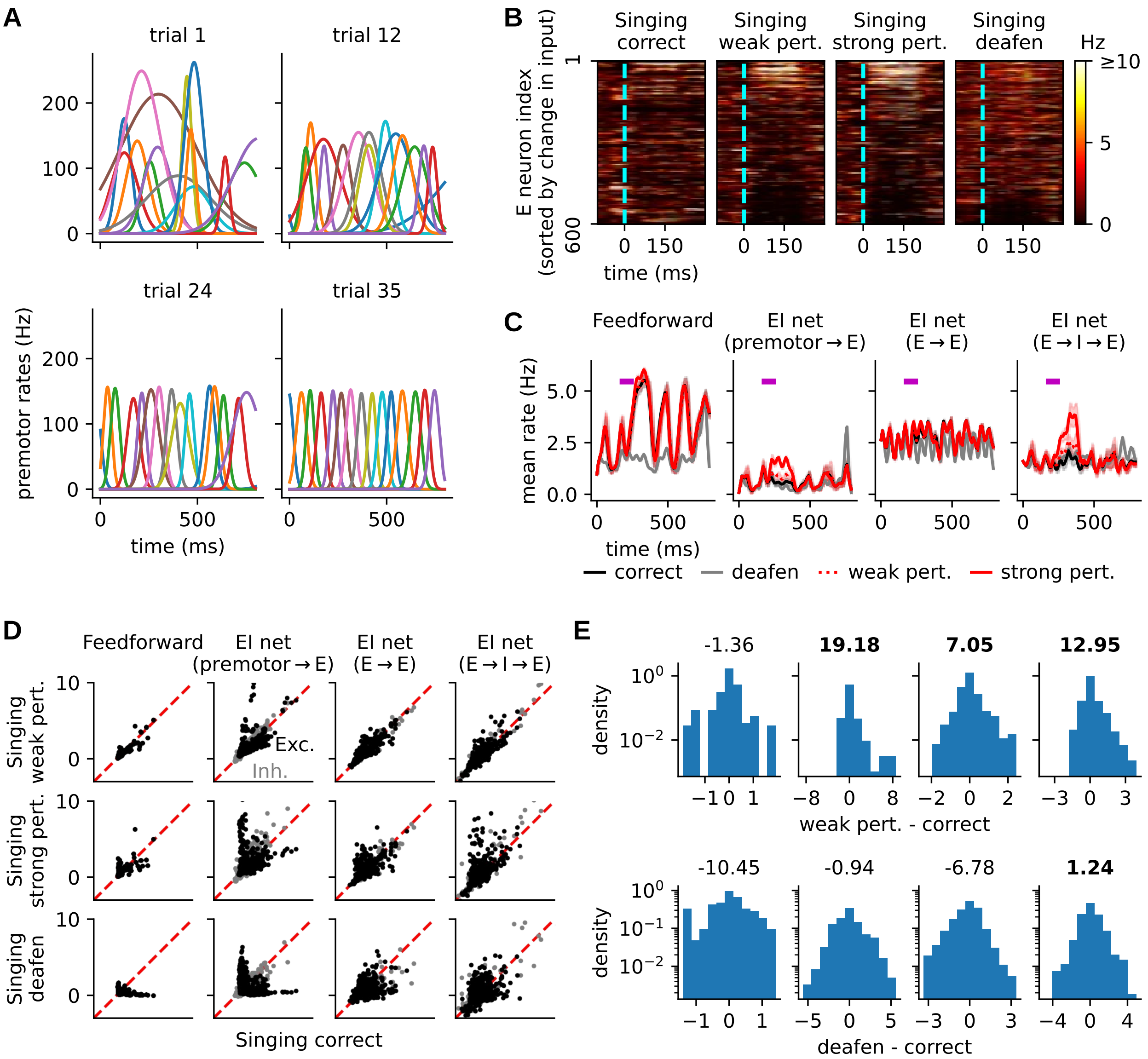

Figure S2: **Learning with gradually maturing premotor profiles.** With less temporally precise premotor input, the feedforward and premotor→E models are even more sensitive to firing thresholds. To see the full range of capabilities for each model, we chose excitatory thresholds $\theta_E = 6$ for the feedforward model, $\theta_E = 4$ for the premotor→E model, and $\theta_E = 0$ for the E→E and E→I→E models. **(A)** During training, the variances of the peak times and peak widths of premotor activities gradually reduce according to a flipped, smooth sigmoidal function of training time (rendition number; see Methods). **(B)** Trained models can represent the changes in auditory feedback compared to tutor song patterns as in Fig. 3D. The heatmaps show the responses of the E→I→E model, but the other models are qualitatively similar. **(C)** As in Fig. 3E, the population mean rates increase following perturbation in the premotor→E and E→I→E models, but not the E→E model. However, different from Fig. 3E, the mean rates in the feedforward model are not different between the correct and perturbation cases. **(D-E)** Only the E→I→E model can stably reproduce the right-skewness in the distributions of change in responses observed in experiments (cp. Fig. 4G).

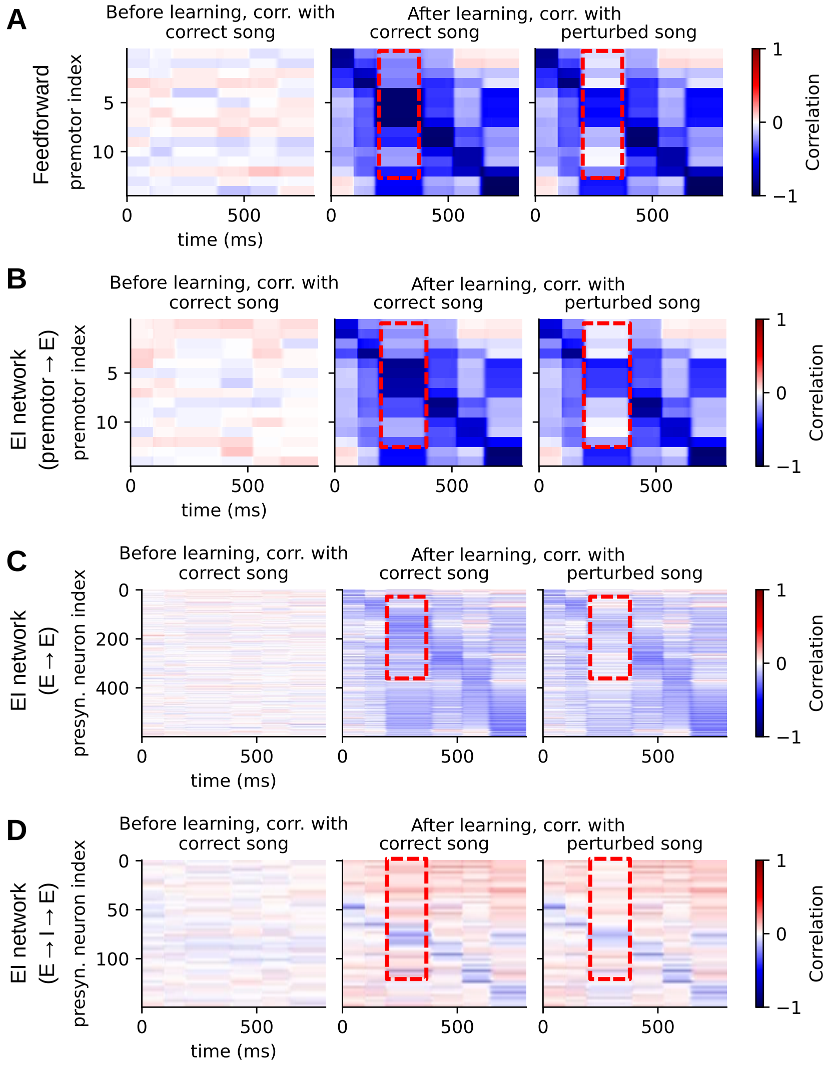

Figure S3: **Correlations between learned weights and sample-averaged tutor song auditory input patterns or perturbed patterns over time.** Left: correlations between the plastic weights and the sample-averaged target song $\mathbf{Y} \in \mathbb{R}^{T \times N_E}$ before training. The plastic weights for calculating the correlations are $\mathbf{W}_E \in \mathbb{R}^{N_E \times N_H}$ (premotor→E connections) in **(A-B)**, $\mathbf{J}_{EE} \in \mathbb{R}^{N_E \times N_E}$ (E→E connections) in **(C)**, and $\mathbf{J}_{EI} \in \mathbb{R}^{N_E \times N_I}$ (I→E connections) in **(D)**. The correlation is calculated between each column of the plastic weight matrix and each row of $\mathbf{Y}$. The middle and right panels are similar, but represent the correlations with the sample-averaged target song after training, and the sample-averaged perturbed song after training, respectively. Red boxes with dashed edges mark the time of perturbation and the synaptic weights that are less correlated with the perturbed patterns than the correct patterns.

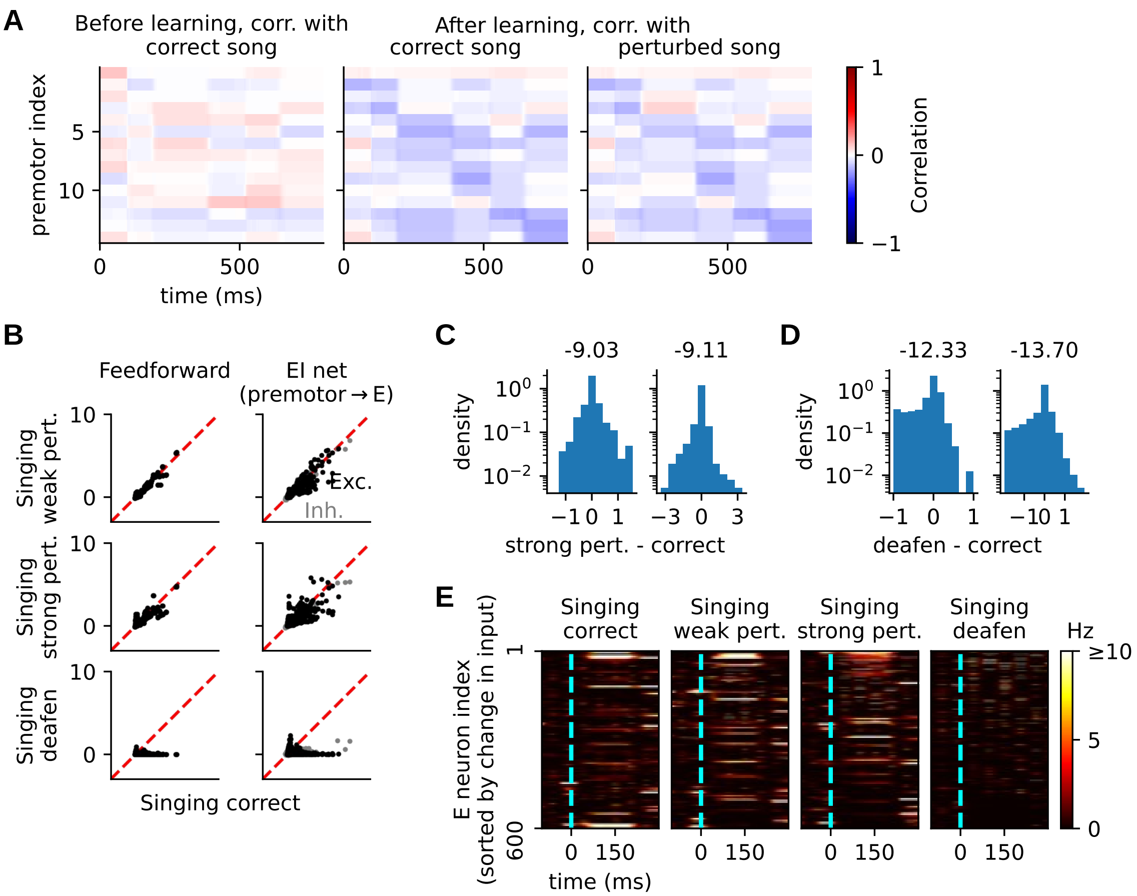

Figure S4: **Sparse premotor projections degrade error signaling in the feedforward and premotor→E models.** The density of premotor→E projection is 10% in this figure. Note that, in contrast, the premotor density is 100% (fully connected) in the feedforward and premotor→E models, and 5% in E→E and E→I→E models in the main results (cp. Fig. 3A-B, Fig. 3D-F, Fig. 4, Fig. 5, Fig. 6). In the case of sparse premotor projections, **(A)** the learned weights and sample-averaged tutor song auditory input patterns or perturbed patterns over time are only weakly correlated and **(B-D)** neurons fail to reproduce the error responses observed in experiments (cp. Fig. 4). **(E)** Only a sparse set of neurons can still represent the difference between the target and perturbed patterns, as shown by the sporadic bright bands, and certain neurons corresponding to smaller mismatch even have the highest firing rates. Neurons during deafened singing are nearly silent, indicating weak predictive coding.

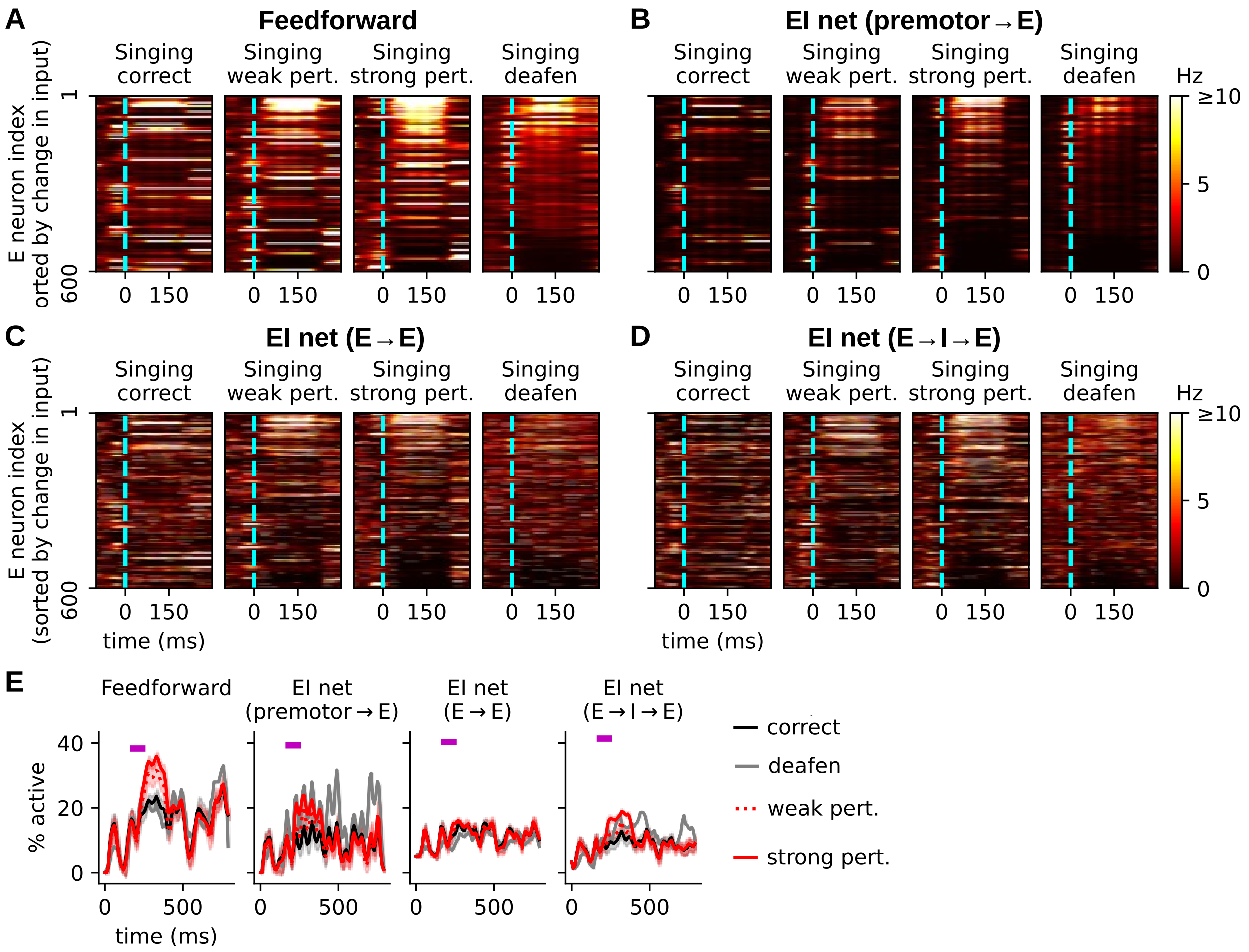

Figure S5: **Heterogeneous error responses in all four models.** **(A-D)** Learned singing responses under normal practice and perturbation in all four models, plotted in the same way as Fig. 3D. Note that Fig. 3D is for the E→I→E model and another example trial is included in **(D)** in this figure for comparisons between models. While the responses are qualitatively similar across models, EI networks display more temporally variable and sparser responses than the feedforward model. **(E)** Percentage of active neurons over time for different models. The purple bar in each subplot indicates the 100-ms white noise perturbation to the auditory feedback of the bird's own song. Following the perturbation, the feedforward, premotor→E, and E→I→ models increase activation density for around 100 ms. The activation density for the E→E model, however, remains stable during white noise perturbation.

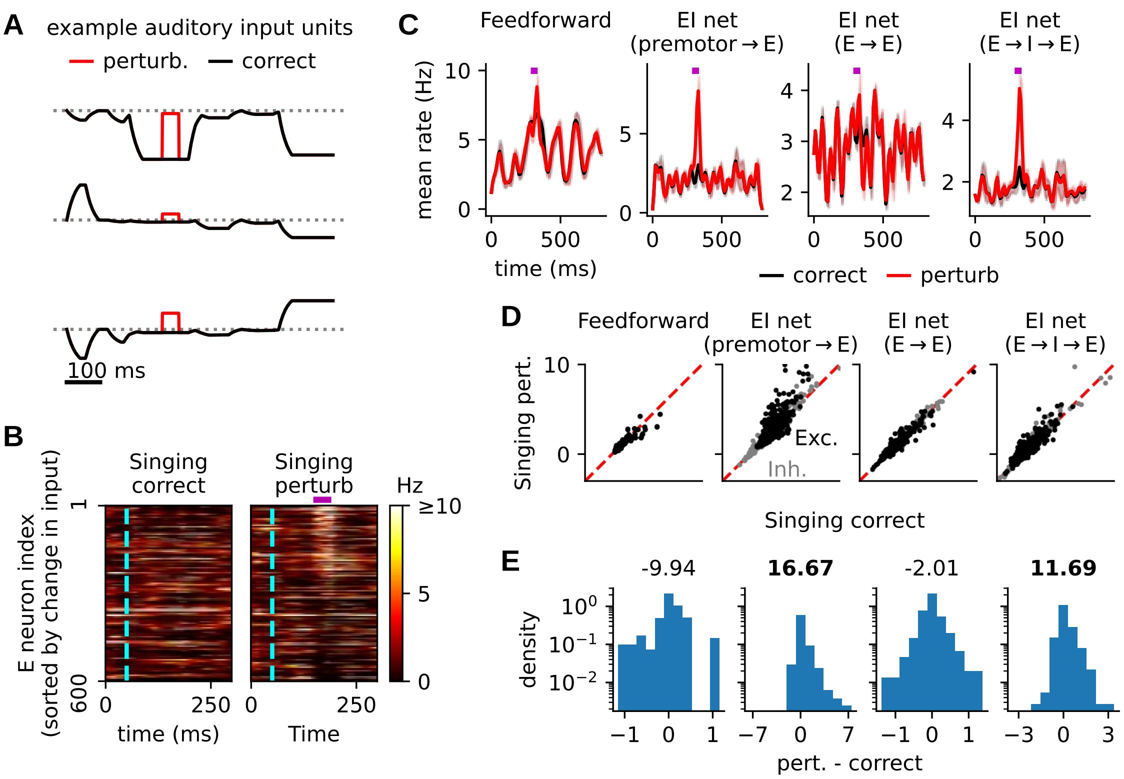

Figure S6: **Models are able to signal perturbation at sub-syllabic level.** **(A)** Three example input units for the correct (black) and perturbed (red) cases. Correct auditory inputs are the sparse representations of the tutor song recordings, as for the experiments in Figs. 3 and 4. The perturbed inputs are constructed by substituting a 50-ms chunk of the correct inputs with a random white noise pattern whose elements are drawn from $\mathcal{N}(0, \sigma^2_{correct})$ where $\sigma^2_{correct}$ is the variance of the correct inputs (red steps in the curves). The 50-ms sub-syllabic perturbation occurs in the middle of a $\sim$150-ms long syllable. **(B)** Learned error responses in the E→I→E model. Cyan dashed lines indicate the start of the perturbed syllable, and the purple horizontal bar indicates the duration of the 50-ms sub-syllabic perturbation. Only shortly (50-100 ms) after the 50-ms sub-syllabic perturbation, the neurons corresponding to larger change in the auditory input patterns have higher firing rates. The other models are qualitatively similar to the E→I→E model. **(C)** Similar to the perturbation at the syllable level (Fig. 3E), the population mean rates in the models except for the E→E model are increased by the sub-syllabic perturbation, but for a much shorter duration. **(D-E)** In agreement with experiments (Fig. 4), sub-syllabic perturbation activates a sparse set of neurons in both premotor→E and E→I→E models, producing right-skewed differential response distributions.

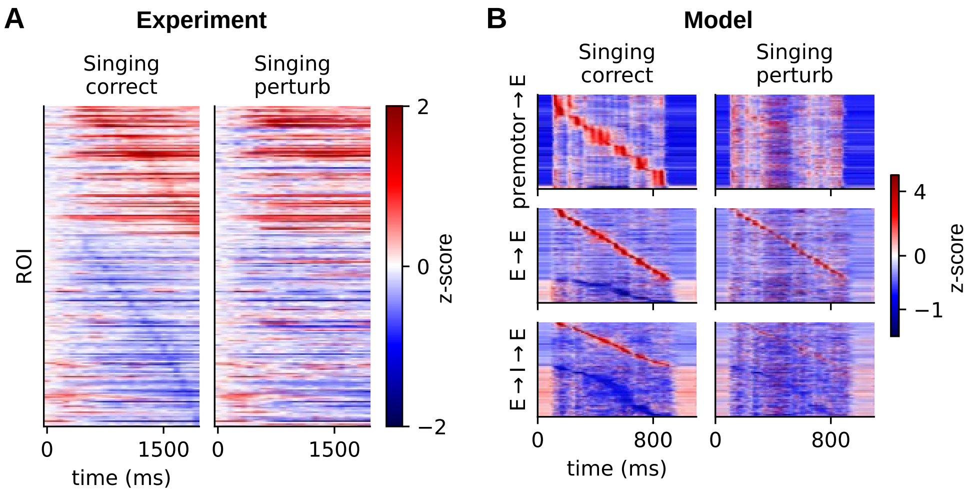

Figure S7: **The E→E and E→I→E models can reproduce both the sequential activation and suppression observed in experiments.** **(A)** Normalized calcium activity in CM during the correct (left) and white noise perturbation (right) cases. In the correct case, neurons display sequential activation or suppression. The order of the sequential activity in the correct singing case is disrupted in perturbation. **(B)** Normalized neuron firing rates for the premotor→E (top), E→E (middle) and E→I→E (bottom) models, during the correct (left) and perturbed (right) singing cases. The feedforward model is qualitatively similar to the premotor→E model. All models are able to reproduce the sequential activation observed in experiments during correct singing. However, only the E→E and E→I→E models can also produce the sequential suppression. The disruption of sequential activity during perturbation is more obvious in the premotor→E and E→I→E models than in the E→E model. Each row of **(A-B)** was standardized using the mean and variance calculated over the displayed time window. In both panels, the neurons in the perturbed singing cases were sorted in the same orders as those in the corresponding correct singing cases.

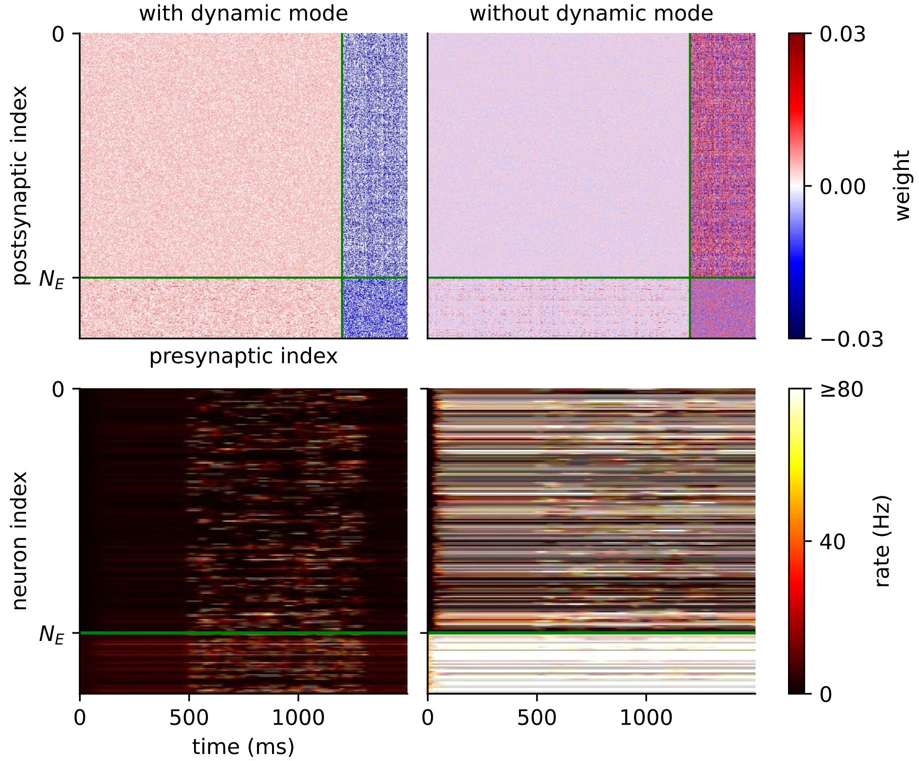

Figure S8: **Removing the dynamic mode in the E→I→E model drastically changes the connectivity and breaks E-I balance.** Top left: original connectivity matrix **J** post-learning. Excitatory and inhibitory synaptic weights are shown in red and blue colors, respectively. Green lines mark the boundary between excitatory neurons and inhibitory neurons. Top right: connectivity matrix post-learning, but with the dynamic mode removed. The removal was done by setting the mode to zero, but the results were qualitatively the same when other perturbation methods were used (see Methods). Bottom left: sparse and mild neuronal firing with the original connectivity weights. Bottom right: dense and hyper-active neuronal firing when the dynamic mode is removed.

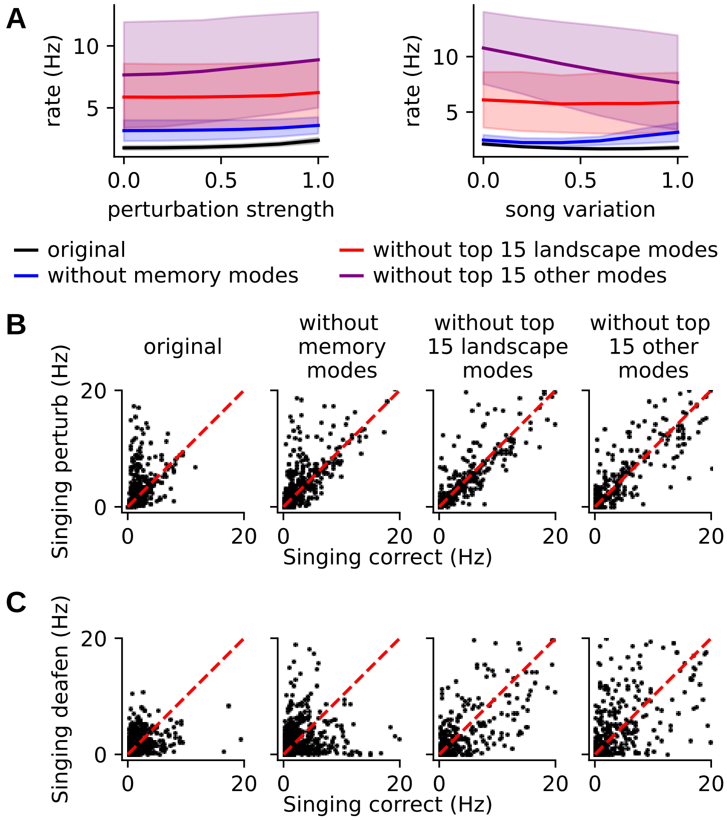

Figure S9: **Connectivity mode perturbations significantly change population activity.** **(A)** Excitatory population mean rates as functions of perturbation strength (left) and song scaling (right) for the original trained models (black); models with top 15 landscape modes removed (red), with memory modes removed (blue); and with top 15 non-memory, non- landscape modes removed (purple). Compared to the original models, all three interventions significantly changed the mean firing rates for every choice of perturbation strength and song scaling ($p < 10^{-5}$, two-sided Wilcoxon rank-sum test). **(B-C)** Distributions of trial-averaged excitatory rates between the correct singing case and perturbed **(B)** or deafened **(C)** conditions for the original trained models (leftmost column) and the three interventions (three right columns).

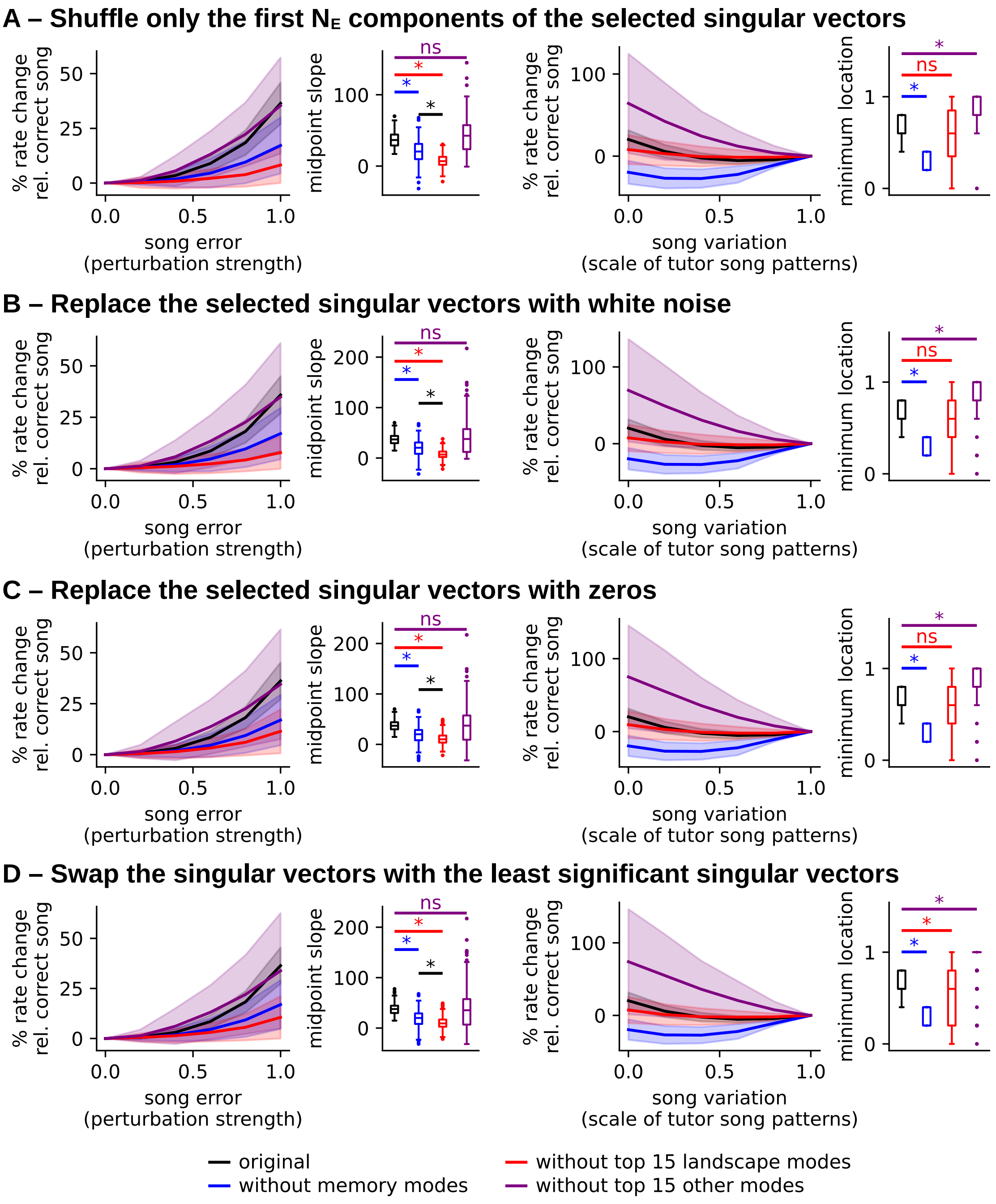

Figure S10: **Perturbing the connectivity modes using alternative approaches.** Each row shows the results from one alternative approach (see Methods) to perturbing the connectivity modes, plotted in the same way as in Fig. 5F-G. All results qualitatively agree with Fig. 5 that perturbing the landscape modes most strongly flattens the error landscape, and only perturbing the memory modes significantly moves the landscape minimum towards the silent input.

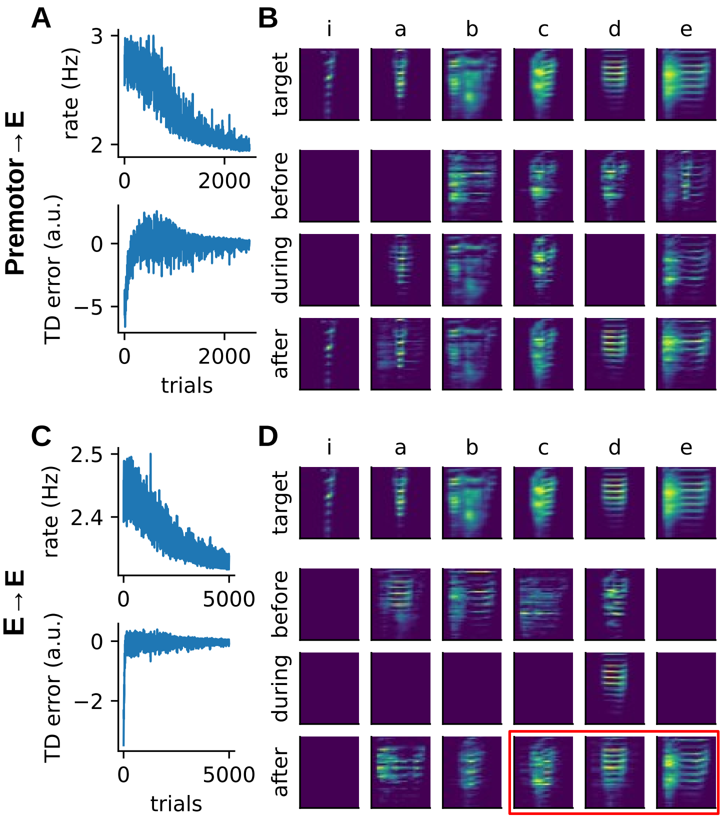

Figure S11: **Error codes produced by the premotor→E and E→E models can also be used to train a motor policy**. Top row, premotor→E model; bottom row, E→E model. **(A)** and **(C)** as Fig. 6B, and **(B)** and **(D)** as Fig. 6C. The red box in **(D)** indicates the generated syllables that match the target syllables.

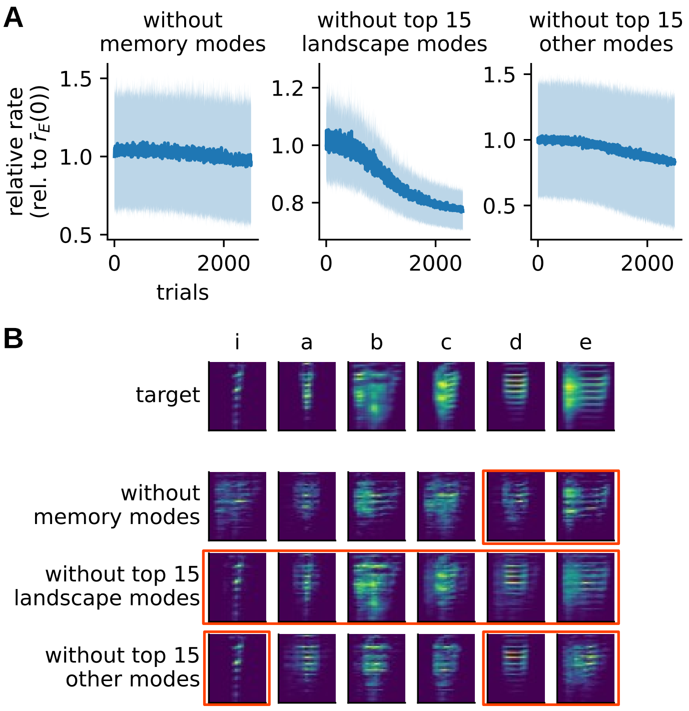

Figure S12: **Syllable generation via RL is impaired when using the error codes from the E→I→E models with perturbed connectivity modes. (A)** The excitatory population rate (error signal) barely decreased over training trials when the memory modes were removed, but decreased robustly in the absence of the top 15 landscape modes. Removal of the top 15 non-memory, non-landscape modes resulted in a slightly decreasing error signal on average, though with very high trial-to-trial variance. Solid curves and the vertical widths of the shaded areas represent the mean and std, respectively, over 12 random initializations. **(B)** Tutor syllable templates (top row), and mean generated syllables after RL using the E→I→E models with different perturbation conditions of the connectivity modes (bottom three rows). The mean was taken over 12 random initializations of training. Taking the median does not qualitative change the results. Red boxes indicate the relatively well-matched syllables. Compared with Fig. 6, the production of many syllables is negatively affected, and more syllables are incorrect in the case with perturbed memory modes than in the other two scenarios.

References

Gao, P., Trautmann, E., Yu, B., Santhanam, G., Ryu, S., Shenoy, K., & Ganguli, S. (2017, November 12). *A theory of multineuronal dimensionality, dynamics and measurement*. https://doi.org/10.1101/214262
